# Regulation of rumen development in neonatal ruminants through microbial metagenomes and host transcriptomes

**DOI:** 10.1186/s13059-019-1786-0

**Published:** 2019-08-23

**Authors:** Nilusha Malmuthuge, Guanxiang Liang, Le Luo Guan

**Affiliations:** Department of Agricultural, Food and Nutritional Science, University of Alberta, Edmonton, Alberta T6G 2P5 Canada

**Keywords:** Neonates, Rumen development, Metagenome, Host transcriptome, Host microRNAome, Host-microbial interactions

## Abstract

**Background:**

In ruminants, early rumen development is vital for efficient fermentation that converts plant materials to human edible food such as milk and meat. Here, we investigate the extent and functional basis of host-microbial interactions regulating rumen development during the first 6 weeks of life.

**Results:**

The use of microbial metagenomics, together with quantification of volatile fatty acids (VFAs) and qPCR, reveals the colonization of an active bacterial community in the rumen at birth. Colonization of active complex carbohydrate fermenters and archaea with methyl-coenzyme M reductase activity was also observed from the first week of life in the absence of a solid diet. Integrating microbial metagenomics and host transcriptomics reveals only 26.3% of mRNA transcripts, and 46.4% of miRNAs were responsive to VFAs, while others were ontogenic. Among these, one host gene module was positively associated with VFAs, while two other host gene modules and one miRNA module were negatively associated with VFAs. Eight host genes and five miRNAs involved in zinc ion binding-related transcriptional regulation were associated with a rumen bacterial cluster consisting of *Prevotella*, *Bacteroides*, and *Ruminococcus*.

**Conclusion:**

This three-way interaction suggests a potential role of bacteria-driven transcriptional regulation in early rumen development via miRNAs. Our results reveal a highly active early microbiome that regulates rumen development of neonatal calves at the cellular level, and miRNAs may coordinate these host-microbial interactions.

**Electronic supplementary material:**

The online version of this article (10.1186/s13059-019-1786-0) contains supplementary material, which is available to authorized users.

## Introduction

The world population is set to reach 9.15 billion by the year 2050, which will increase demand for food, particularly the demand for animal proteins [1]. Ruminants (cattle, sheep, goat) are physically distinguishable from monogastric animals due to the presence of forestomach (rumen, reticulum, omasum) and play a vital role in meeting high-quality animal protein (meat and milk) production demand all over the world. The rumen is the unique organ of ruminants that converts low-quality forage into high-quality animal protein through microbial fermentation. Rumen fermentation is a complex process conducted by the symbiotic microbiota, which produces 70% of the ruminant’s daily energy in the form of volatile fatty acids (VFAs) [2]. Manipulation of the rumen microbiota is one of the potential approaches to enhancing rumen fermentation [3]. However, the current understanding of the establishment of the rumen microbiome and its importance for rumen development remains very limited, which is a barrier to achieving such improvement.

Ruminants are born with underdeveloped rumen, reticulum, and omasum and are considered functionally monogastric animals before weaning [4]. Neonatal ruminants (does not yet chew the cud; pre-ruminants) undergo physiological changes in the rumen before they can solely depend on fiber-rich diets [4]. The development of the rumen, which facilitates a smooth weaning transition from pre-ruminant to ruminant [4], has mainly been studied during weaning itself. This process is influenced by the calf’s diet [5, 6], the feeding methods [7], and the microbial colonization [8]. Recently, an increasing number of studies have explored the molecular mechanisms underlying rumen development during the weaning transition [9, 10] as well as the rumen microbiota in pre-ruminants [11–14]. Rumen microbial colonization begins as early as the first day of life [12], and the pre-weaning diet alters its composition and the production of VFAs [15], suggesting the importance and the potentials of pre-weaning feeding interventions to manipulate the early rumen microbiota to alter rumen development. Nevertheless, the mechanisms regulating early rumen development process, especially the role of microbiota, are largely unknown.

Our previous studies revealed the establishment of rumen-specific bacteria [13] as well as the presence of rumen-specific microRNA (miRNA, a group of non-coding RNAs) profiles associated with the bacterial densities [16] in pre-ruminants. Thus, this study hypothesized that the early microbiome is actively involved in rumen development through its interaction with the host transcriptome. We employed next-generation sequencing of the rumen microbial metagenomes and rumen tissue transcriptomes (RNA-seq sequencing of host mRNAs and microRNAs) with an integrated bioinformatics approach to explore host-microbial interactions and their roles in regulating rumen development in pre-ruminants. Further, we evaluated the establishment and functionality of early rumen microbiota via quantification of active microbial densities (RNA-based) and VFA (acetate, butyrate, propionate, branched-chain FAs) production. A detailed understanding of early rumen development (functions, morphology, and colonization) may provide a mean to manipulate its functions in the future to improve the productivity and health of ruminants and to meet global food production demands.

## Results

### Active and functional microbiota establishes at birth

**Fig. 1:**
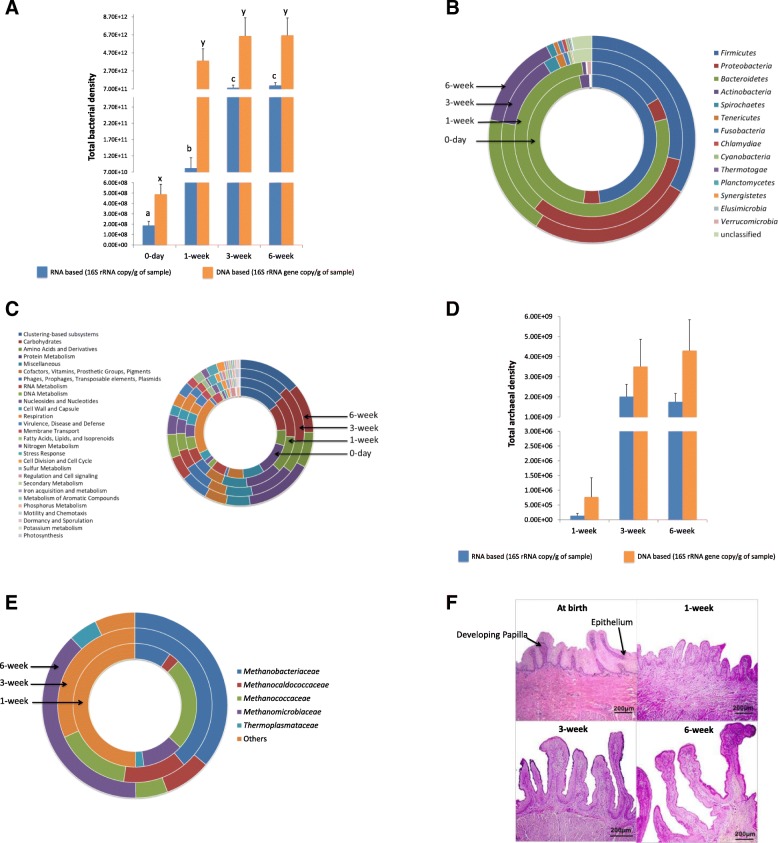
Establishment of rumen microbiome from birth up to the first 6 weeks of life and the development of rumen papillae. **a** Estimated total bacterial density (DNA-based (16S rRNA gene copy/g of sample) and RNA-based (16S rRNA copy/g of sample)) in calf rumen during the first 6 weeks of life (*P* = 0.02). Bars represent mean bacterial densities, and error bars represent SEM. a and b represent the mean RNA-based bacterial densities different at *P* < 0.05. x and y represent the mean DNA-based bacterial densities different at *P* < 0.05. **b** Composition of rumen content-associated bacteria (mean relative abundance) at the phylum level. **c** Functional composition of rumen content-associated bacteria at level 1 SEED hierarchy/subsystems. **d** Estimated total archaea density using DNA-based (16S rRNA gene copy/g of sample) and RNA-based (16S rRNA copy/g of sample) quantifications. **e** Rumen content-associated archaeal composition at the family level. **f** Rumen papillae development in calves within the first 6 weeks of life. Images are obtained through a light micrograph of rumen tissue at a magnification of × 10 objective lens (bar = 200 μm)

We used a metagenomics-based approach together with DNA and RNA-based quantification (quantitative PCR) of microbiota to explore the calf rumen microbial colonization from birth up to 6 weeks of life. The use of metagenomics-based sequencing revealed that the newborn calf rumen was mainly colonized with a diverse (83 genera, Additional file 1) bacterial community (99.9 ± 0.5%) at birth (Additional file 2: Figure S1). No archaea and protozoa were detected in the calf rumen at birth, while fungi and viruses together accounted for ~ 0.1% of total identified rumen microbiota (Additional file 2: Figure S1). The use of qPCR analysis further revealed initial bacterial colonization was dense (9.1 ± 3.1 × 10^8^ 16S rRNA gene copy/g) and active (1.9 ± 0.4 × 10^8^ 16S rRNA copy/g) (Fig. 1a). *Veillonella*, followed by *Prevotella*, *Bacteroides*, *Eubacterium*, *Streptococcus*, *Acidaminococcus*, *Clostridium*, *Bifidobacterium*, and *Ruminococcus*, were predominant (account for 88.7%) in the calf rumen at birth (Additional file 1). The abundance of the other identified 72 genera accounted for only 11.3% of the rumen bacteria. Microbial function assignment using the SEED subsystems hierarchy (subsystems hierarchy—the collection of related functional roles represented in four-level hierarchy) revealed 27 level 1 (level 1—the highest level of subsystem, e.g., protein metabolism) and 116 level 2 (subpathways within a major metabolic pathway, e.g., protein biosynthesis) functions along with 543 microbial genes (level 4) at birth. The predominant subsystems identified in the calf rumen were “respiration” and “protein metabolism” (Additional file 1), whereas “folate and pterines” (11.2 ± 2.3%) and “electron donating (9.1 ± 0.5%) and accepting” (5.3 ± 0.6%) functions were prevalent among the level 2 functions. The predominant microbial genes identified at birth were “decarboxylase” (8.6 ± 7.7%) and “NADH dehydrogenase” (4.7 ± 4.3%).

### Rumen microbiome undergoes rapid changes during early life

Metagenomics analysis also showed that the rumen of pre-weaned calves (1-week, 3-week, and 6-week) was colonized by bacteria, archaea, protozoa, fungi, and viruses (Additional file 2: Figure S1), while bacteria remained predominant. The bacterial density in the calf rumen increased 438-fold (RNA-based; *P* < 0.05) and 7829-fold (DNA-based; *P* = 0.02) within the first week of life (Fig. 1a). The identified bacteria belonged to 14 different phyla, dominated by *Firmicutes*, *Bacteroidetes*, *Proteobacteria*, and *Actinobacteria* (Fig. 1b, Additional file 1). A total of 167 genera identified, with 9.3 ± 2.2% unassigned sequences, 63 of which were predominant bacterial genera (abundance > 1% in at least 1 sample). Among the detected genera, *Prevotella*, *Bifidobacterium*, *Corynebacterium*, *Streptococcus*, *Lactobacillus*, *Clostridium*, *Staphylococcus*, *Bacillus*, *Campylobacter*, *Pseudomonas*, *Yersinia*, *Neisseria*, *Campylobacter*, *Haemophilus*, *Burkholderia*, *Vibrio*, and *Brucella* were present in all the pre-weaned calves. The prevalence of the identified bacterial genera varied with the calf age, with substantial differences observed when comparing week 1 against weeks 3 and 6 (Additional file 1). For example, the abundance of *Prevotella* in the microbial metagenome was higher (*P* < 0.05) in week 1 than weeks 3 and 6 (Additional file 1); however, the qPCR-based density of active *P. ruminicola* increased numerically (*P* > 0.1) with calf age (Table 1). A higher prevalence (*P* < 0.05) of *Ruminococcus* was observed from the first week of life in the rumen microbial metagenome (Additional file 1). RNA-based quantification also revealed the colonization of both *R. flavefaciens* and *R. albus* in the rumen from the first week (Table 1). Only active *R. flavefaciens* increased significantly (*P* = 0.03) with increasing age, while *R. albus* (*P* = 0.34) increased numerically (Table 1). The prevalence of *Eubacterium* and *Roseburia* in the rumen microbial metagenome also increased (*P* < 0.05) with increasing age (Additional file 1), with the introduction of solid feed. For example, the abundance of *Eubacterium* and *Roseburia* increased by 12- and 86-fold, respectively, from week 1 to week 6. However, there were no significant temporal changes in the active *E. ruminantium* density (Table 1).

**Table 1:** Postnatal changes in active rumen bacteria, rumen morphology, and metabolites of pre-weaned calves

	Calf age			*P* value
	1 week	3 weeks	6 weeks	
Active rumen bacteria				
*R. flavefaciens*^1^	3.8 ± 1.9E08^a^	1.6 ± 0.4E09^b^	1.3 ± 0.5E09^b^	0.03
*R. albus*	1.4 ± 0.5E05	6.3 ± 6.2E06	2.2 ± 1.7E07	0.34
*P. ruminicola*	6.4 ± 2.7E04	4.4 ± 3.0E05	7.6 ± 7.4E07	0.37
*E. ruminantium*	7.8 ± 3.8E05	1.5 ± 0.6E06	1.6 ± 0.6E06	0.48
Rumen papillae				
Length (μm)	317.8 ± 7.6^a^	413.0 ± 13.6^b^	678.1 ± 41.1^c^	< 0.01
Width (μm)	155.5 ± 2.7^a^	224.0 ± 6.3^b^	275.8 ± 9.0^c^	< 0.01
Rumen fermentation parameters				
Acetate^2^	21.1 ± 2.2^a^	37.0 ± 2.8^b^	50.3 ± 3.8^c^	< 0.01
Propionate	10.6 ± 2.0^a^	25.8 ± 4.3^b^	36.3 ± 1.8^c^	< 0.01
Butyrate	5.6 ± 1.8^a^	11.8 ± 2.7^b^	17.8 ± 2.2^b^	< 0.01
Isobutyrate	0.3 ± 0.04^a^	0.8 ± 0.1^b^	1.3 ± 0.1^c^	< 0.01
Valerate	1.0 ± 0.4^a^	3.7 ± 0.7^b^	5.1 ± 0.8^b^	< 0.01
Isovalerate	0.3 ± 0.07^a^	0.8 ± 0.1^a^	1.3 ± 0.3^b^	< 0.01
Total	39.6 ± 6.2^a^	80.5 ± 9.0^b^	113.9 ± 6.9^c^	< 0.01
Acetate^3^	55.3 ± 2.7^a^	47.0 ± 2.4^b^	44.2 ± 1.9^b^	0.01
Propionate	26.7 ± 1.4	31.3 ± 2.9	32.1 ± 1.4	NS
Butyrate	13.0 ± 2.0	14.4 ± 2.5	15.4 ± 1.4	NS
Isobutyrate	0.8 ± 0.1	1.0 ± 0.1	1.2 ± 0.1	NS
Valerate	2.1 ± 0.5^a^	4.6 ± 0.6^b^	4.5 ± 0.6^b^	0.01
Isovalerate	0.8 ± 0.2	1.0 ± 0.2	1.2 ± 0.2	NS

Means with different superscript letters within a row are significantly different at *P* < 0.05

^1^Density of active rumen bacteria (16S rRNA copy/g of rumen content)

^2^VFA concentration (mM/mL of rumen fluid)

^3^Molar proportion of VFA (%)

In total, 28 level 1 and 168 level 2 functions in the SEED subsystems hierarchy were observed in pre-weaned calves (from week 1 to week 6). Among these, the subsystems related to “protein and carbohydrate metabolism” dominated the rumen microbiome (Fig. 1c, Additional file 1). “Protein metabolism” mainly consisted of microbial functions related to “protein biosynthesis,” while “carbohydrate metabolism” comprised microbial functions related to “central carbohydrate metabolism” at level 2 of the SEED subsystems hierarchy. The differentially abundant microbial genes were mainly identified when comparing week 1 calves against week 3 and week 6 calves (Additional file 1). In total, 3443 microbial genes were identified from all pre-weaned calves but with a high inter-individual variation. The majority of differentially abundant microbial genes were observed between weeks 1 and 6 (396), followed by weeks 1 and 3 (134) and week 3 and 6 (59). Nineteen microbial genes encoding glycoside hydrolases (GHs) were identified in the pre-weaned rumen microbiome with varying relative abundance over calf age (Additional file 1). The abundances of α-galactosidase, α-glucosidase SusB, α-l-arabinofuranosidase II precursor, α-*N*-acetylglucosaminidase, α-*N*-arabinofuranosidase 2, β-galactosidase large subunit, glucan 1,6-alpha-glucosidase, and maltose-6′-phosphate glucosidase were higher in week 6 than in weeks 1 and 3 (Additional file 1).

### Active archaea established in neonatal calves from the first week of life

Quantification of 16S rRNA gene using RNA-based real-time PCR revealed the colonization of active archaea from the first week of life (Fig. 1d), while the archaeal density was 10,000-fold lower (*P* < 0.01) in week 1 compared to weeks 3 and 6 (Fig. 1d). Similarly, metagenomics-based sequencing revealed archaeal colonization from the first week of life (0.03 ± 0.01%) that increased relative abundance by 41- and 54-fold in weeks 3 and 6 calves, respectively. Regardless of the presence of archaea from the first week, methyl coenzyme M reductase gene (mcrA) was only detected in the microbial metagenomes of weeks 3 (0.2 ± 0.0003%) and 6 (0.2 ± 0.0001%) calves. A higher abundance of microbial genes encoding archaeal-specific glycolysis enzymes (glucose-6-phosphate-isomerase, fructose-biphosphate aldolase, 2,3-biphosphate-independent phosphoglycerate mutase, and non-phosphorylating glyceraldehyde-3-phosphate dehydrogenase) was observed in week 1, compared to weeks 3 and 6 (Additional file 1). Metagenomics sequencing further revealed that the pre-ruminant ruminal archaea mainly consisted of the families *Methanomicrobiaceae*, *Methanobacteriaceae*, and *Methanococcaceae* (Fig. 1e). The prevalence of *Methanobacteriaceae* observed in microbial metagenomic profiles was higher (*P* = 0.01) in weeks 3 (39.0 ± 9.8%) and 6 (36.1 ± 14.3%) than week 1 (9.6 ± 6.0%). Although no single genus was present in all the calves, *Methanobrevibacter*, *Methanothermobacter*, *Methanobacterium*, and *Methanoplanus* were observed in 60% of week 6 calves.

### Rumen epithelium development and VFA profile in pre-weaned calves

The rumen epithelium at birth displayed a unique structure compared to pre-weaned calves (Fig. 1f). There were no separated protruding papillae or stratified squamous epithelium in the calf rumen soon after birth; however, developing papillae were noticeable (Fig. 1f). The rumen epithelium of newborn calves was consisted of a large number of nucleated squamous cells with a thickness of 279.9 ± 7.6 μm that later developed into 678.1 ± 41.1 μm length papillae within 6 weeks. The increase in the length and width of the rumen papillae was significantly different among the three age groups (Table 1).

The concentration of total VFA, acetate, butyrate, propionate, valerate, isobutyrate, and isovalerate increased with increasing age and dietary changes (Table 1). However, only the molar proportion of acetate and valerate displayed age-related variations, while the molar proportion of butyrate ranged from 13 to 16% of total VFA during the first 6 weeks of life (Table 1). In addition, the concentration of VFAs was positively correlated with active *R. flavefaciens* density and the rumen papillae development (Additional file 2: Table S1).

### Microbiome-host transcriptome interactions may influence rumen epithelial development and tissue metabolism

**Fig. 2:**
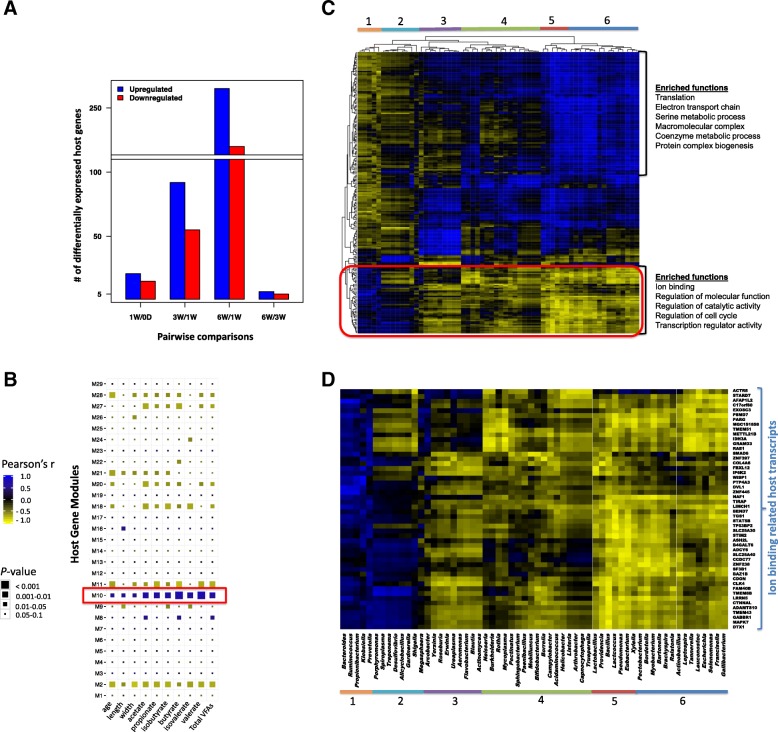
Associations among the transcriptome networks (gene modules), calf phenotypic traits (concentration of VFAs, papillae length and width, calf age) and bacterial composition (taxonomy—genus level). **a** Number of differentially expressed genes between each pairwise comparison during postnatal period. **b** Relationship between gene modules (gene modules are defined as M1–M29) and calf phenotypic traits. Gene modules obtained using weighted gene co-expression network analysis and eigengene/PC1 value of each gene module is correlated with the calf phenotypic traits. **c** Association between the host genes co-expressed in the M10 module and rumen content-associated bacterial genera relative abundance. **d** Bacterial clusters associated with ion binding-related genes co-expressed in the M10 module. Cluster 1 (*Bacteroides*, *Ruminococcus*, *Propionibacterium*, *Klebsiella*, *Prevotella*) positively correlates to the expression of the ion binding-related genes (*P* < 0.05, *r* ≥ 0.5). Cluster 6 (*Pectobacterium*, *Bordetella*, *Mycobacterium*, *Bartonella*, *Brachyspira*, *Ralstonia*, *Actinobacillus*, *Leptospira*, *Tannerella*, *Leuconostoc*, *Escherichia*, *Selenomonas*, *Francisella*, *Gallibacterium*) negatively correlates to the expression of the ion binding-related genes (*P* < 0.05, *r* ≤ − 0.5). Heatmap is generated using Pearson’s correlation value between the expression of a gene and the relative abundance of a bacterial genus. Blue represents positive correlations, whereas yellow represents negative correlations. Numerical values represent the identified bacterial clusters based on their associations with the expression of genes

Host-microbial interactions in the developing rumen were evaluated via identifying the associations among rumen transcriptomes, the papillae length and width, the concentration of VFAs, and the microbial metagenomes (composition and functions). RNA-seq-based transcriptome profiling (total mRNA sequencing) revealed a total of 13,676 ± 399 genes (CPM > 1) expressed in the calf rumen tissue. A higher number of differentially expressed (DE) genes were observed when comparing between newborn (0-day) and 1W calves (36) and 1W and 3W calves (147), but not between 3W and 6W calves (7) (Fig. 2a; Additional file 3). The use of weighted gene co-expression network analysis (WGCNA) clustered the common host genes (11,772; Additional file 3) expressed in all calves into 29 gene modules (defined as M1–M29 modules; Fig. 2b, Additional file 2: Figure S2). These gene modules displayed various associations with the calf phenotypic traits (papillae length and width, the concentration of VFAs—acetate, butyrate, propionate, branched-chain FAs, and total, calf age). The expression of host genes in the M2 module (2313 genes; 13.8% of total reads) and M18 module (212 genes, 0.95% of total reads) was negatively correlated, while the expression of genes in the M10 module (1070 genes, 22.5% of total reads) was positively correlated with calf phenotypic traits (Fig. 2b, Additional file 2: Figure S2). Host genes co-expressed in the M2 module were related to “transcription,” “splicing,” “ribonucleoprotein complex biogenesis,” and “RNA metabolic process” (Additional file 2: Figure S2). Host genes co-expressed in the M18 module were enriched with functions related to “chromatin organization,” “histone modification,” and “transcription” (Additional file 2: Figure S2). Histone genes (*H1F0*, *H1FX*) and histone deacetylase coding genes (*HDAC3*) co-expressed among the 9 host genes involved in “chromatin organization.” Host genes co-expressed in the M10 module involved in “tissue metabolism-related” functions (Additional file 2: Figure S2, Additional file 4), and the largest proportion of these genes (38 genes, 7.65% of total reads) related to “respiratory electron transport chain” (Additional file 2: Figure S3). They consisted of “mitochondrial respiratory chain complex proteins,” such as “cytochrome c oxidase subunits” (*COX1*, *COX3*, and *COII*), “NADH dehydrogenase subunits” (*ND2*, *ND5*), “succinate dehydrogenase subunits,” “ubiquinol-cytochrome c reductase subunits,” and “ATP synthase subunits” (Additional file 2: Figure S3).

The M10 module, which clustered host genes related to “rumen tissue metabolism” and positively correlated with the concentration of VFAs (total, acetate, butyrate, propionate, and branched-chain FAs), was subjected to further analysis to explore the role of bacteria in early rumen development. Clustering of the correlation coefficient between the gene expression and the relative abundance of bacterial genera revealed 6 bacterial clusters depend on their association patterns (Fig. 2c). A cluster (cluster 1) consisting of *Prevotella*, *Bacteroides*, *Ruminococcus*, *Klebsiella*, and *Propionibacterium* was positively correlated with the expression of 49 host genes involved in “ion binding”; “regulation of cell cycle, catalytic activity, molecular functions”; and “transcription regulatory activity” (Fig. 2c). The majority of “ion binding” host genes (8/13) were related to zinc finger proteins (*ZNF*s) (LIM and calponin homology domains1, *ZNF238*, *ZNF445*, *ZNF397*, bromodomain adjacent to zinc finger domain1B, ADAM metallopeptidase with thrombospondin type 1 motif 10, deltex 1 E3 ubiquitin ligase, ash2 (absent, small, or homeotic)-like). Another cluster (cluster 6) containing genera mainly from *Firmicutes* and *Proteobacteria* was negatively correlated with the expression of the same set of genes (Fig. 2d).

**Fig. 3:**
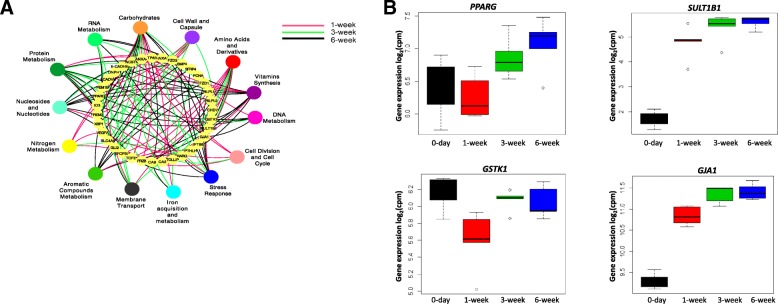
**a **Level 2 microbial functions associated with (*P* < 0.01, *r*^2^ ≥ 0.98) host genes involved in rumen epithelial tissue development (GO: 0060429, 34 genes). **b** Level 2 microbial functions associated genes co-expressed in M10 gene module. *PPARG* – peroxisome proliferator activated receptor gamma; *SULT1B1* – sulfotranferase family 1B member 1;* GSTK1* – glutathione S-transferase kappa 1; *GJA1* – gap junction protein alpha 1. 0-day – at birth, 1-week – 1-week-old calves, 3-week – 3-week-old calves, 6-week – 6-week-old calves

Among the level 2 microbial functions, “microbial carbohydrate metabolism” was strongly linked to the expression of host genes. Among these correlated host genes, there were 19 of 34 genes related to “rumen epithelium development” (Fig. 3), “rumen tissue carbohydrate metabolism” (Additional file 2: Figure S4), and “membrane transportation” (solute carrier family 35 and monocarboxylate transporters—*SLC16A3/MCT3*, *SLC16A9/MCT9*, *SLC16A11/MCT11*, *SLC16A13/MCT13*) (Additional file 2: Figure S4) as well as 8 of 14 “tight junction protein genes” (TJs) (Additional file 2: Figure S5). Some of these microbial carbohydrate metabolism-associated host genes were co-expressed in the M10 module, such as *FUCA1*, *GANC*, *GALC* (related to “rumen tissue carbohydrate metabolism”; Additional file 2: Figure S4B), *SLC35A3* (related to “membrane transportation,” Additional file 4: Figure S4C), *CLDN23* (related to TJs; Additional file 2: Figure S5), and *PPARG*, *GSTK1*, *SULT1B1*, and *GJA1* (related to “rumen epithelial development”; Fig. 3).

### microRNAome coordinates microbiome-host transcriptome crosstalk

**Fig. 4:**
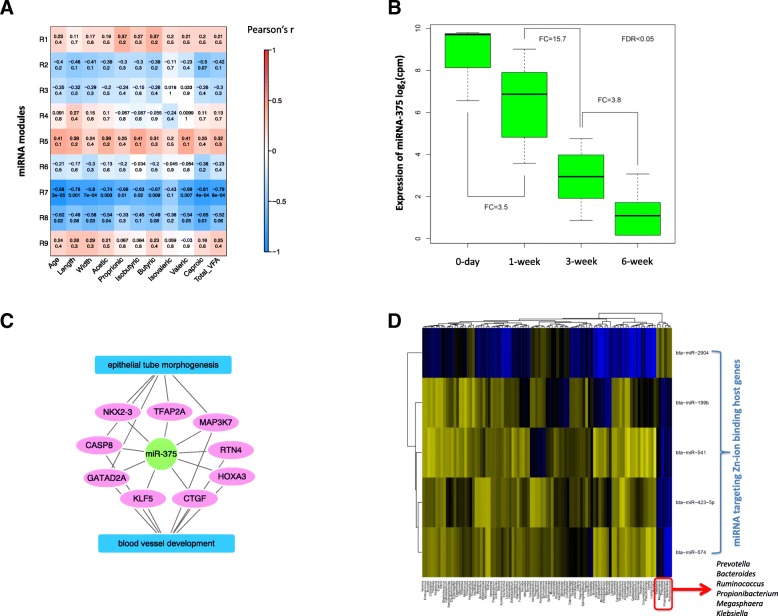
Association between rumen miRNA profile (expression of miRNA) and rumen microbiota (bacterial genera, concentration of VFAs). **a** Relationship between the miRNA modules (miRNA modules define as R1–R9) and calf phenotypic traits. miRNA modules are generated using WGCNA, and eigengene/PC1 values of each modules are correlated with calf phenotypic traits. Numerical values within a square represent Pearson correlation (upper value) and *P* value (lower value). Color bar represents Pearson correlation from − 1 to 1. **b** Temporal changes in the expression (CPM) of miR-375 in calf rumen (day 0, 605.1 ± 40.3; week 1, 171.5 ± 15.6; week 3, 10.9 ± 3.8; week 6, 2.9 ± 1.2; *P* < 0.01). Fold change (FC) is the expression ratio between two adjacent age groups. **c** Functions of mir-375 predicted using TargetScan and miRbase. **d** Association between rumen bacterial taxonomy and miRNAs co-expressed in the R7 miRNA module

To identify potential regulatory mechanisms of host-microbial interactions, microRNAome data (364 ± 17 miRNAs) generated using the same animals in a previous study [16] were analyzed using WGCNA to identify their relationships with calf phenotypic traits (papillae length and width, the concentration of VFAs—acetate, butyrate, propionate, branched-chain FAs, and total, calf age). The rumen microRNAome was clustered into 9 modules (defined as R1–R9 miRNAs modules) based on the co-expression of miRNAs (Fig. 4a). The R7 miRNA module (129 miRNAs) was negatively correlated with the calf phenotypic traits and the concentration of VFAs, except isovalerate (Fig. 4a). The use of targetScan and mirBase revealed miRNAs co-expressed in R7 had 3710 predicted genes in total. Among the R7-predicted genes, 3847 (~ 96%) were expressed in the rumen tissue transcriptome of the present study. Moreover, 258 of the predicted 3710 were co-expressed in the M10 module identified from the rumen tissue transcriptome. Temporally downregulated R7 member miR-375 (Fig. 4b) was involved in “rumen epithelial morphogenesis-” and “blood vessel development-related” functions (Fig. 4c, Additional file 5). The R8 miRNA module (40 miRNAs) was also negatively correlated with the calf age, papillae width, acetate, and valerate (Fig. 4a). The miRNAs co-expressed in the R8 module had 2751 predicted target genes in total, and 2649 (~ 96%) of these genes were expressed in the calf rumen tissue transcriptome of the present study. Functional analysis revealed that miRNAs co-expressed in the R8 module were involved in “protein localization and transportation” and “cell motility” (Additional file 5). However, only R7 miRNAs had their targets co-expressed in the M10 module.

The roles of miRNAs in regulating host-microbial interactions were further evaluated via exploring the relationships among the expression R7 miRNAs, M10 genes, and the relative abundance of bacterial genera. Nearly 37% (55/147) of the M10 genes associated with bacterial clusters 1 and 6 (Fig. 2d) were targeted by 28 miRNAs co-expressed in R7. Among these, bta-miR-2904, bta-miR-199b, bta-miR-541, bta-miR-574, and bta-miR-423-5p were associated with a bacterial cluster comprising *Prevotella*, *Bacteroides*, *Ruminococcus*, *Propionibacterium*, *Klebsiella* (cluster 1 from Fig. 2d), and *Megasphaera* (Fig. 4d). Furthermore, these 5 miRNAs targeted 65 different genes related to *ZNF*s identified in the host transcriptome (Additional file 5).

## Discussion

The microbiota that rapidly colonizes the in utero sterile mammalian gut during and after birth constantly interacts with the host to maintain metabolism and health. The early gut microbiome has been suggested to have a long-term impact on human health [17]. Despite the accumulating knowledge on the diversity of the rumen microbiome during early life [11–14, 18], the importance of rumen colonization for tissue development and the regulatory mechanisms of host-microbial interactions in pre-ruminants are largely unknown.

This study revealed the establishment of a dynamic, dense, and active microbiome in the pre-ruminant rumen at birth that undergoes rapid changes during the first 6 weeks of life using microbial metagenomics sequencing and RNA-based microbial quantification. The gut microbiota has been widely studied in mammalian species using DNA-based approach; however, it is evident that such evaluation may overestimate both the organisms and their activities. The RNA-based quantification used in this study revealed the colonization of active bacteria within a few minutes of birth, indicating that the process might have started during the birthing process, which extended from an hour to 3 h. Exploring the dam birth canal (*Streptococcus*, 23.3 ± 13.3%; *Ruminococcaceae*, 12.6 ± 4.6%) and rectal bacteria (*Ruminococcaceae*, 18.9 ± 1.8%) following birth (data not shown) suggested that the vaginal/fecal bacteria of dams were the main inoculum of the calf rumen bacteria at birth. Our findings also confirmed previous studies claiming the establishment of fibrolytic bacteria within the first week of life [18], a higher prevalence of *Prevotella* [11, 14], and the presence of GHs in the absence of proper substrates [11]. We revealed colonization with active *R. flavefaciens*, *R. albus*, *E. ruminantium*, and *P. ruminicola*, the classical rumen bacteria that degrade plant polysaccharides (cellulose, hemicellulose, xylan and glycan) [19, 20], from the first week of life, when calves were fed solely with milk. The increasing density of these species coincided with elevated concentration of VFAs as well as increased papillae length and width of week 3 and 6 calves fed starter and milk. This finding suggests that the introduction of a solid diet stimulates the rapid growth of the rumen papillae by influencing the rumen microbial composition and functions. Traditionally, solid feed is considered the major driver of rumen development, which stimulates microbial fermentation [4, 9]. However, the appearance of cellulolytic bacteria [18] and the activity of xylanase and amylase [21] can be detected from the second day of life. Thus, we propose that the presence of active microbiome as early as the first week calls for a detailed understanding of their roles in the development of the rumen.

The removal of H_2_ from the rumen, which has inhibitory effects on microbial fermentation, increases the rate of fermentation [22] and can be considered as one of the features of rumen development. The presence of mcrA gene in the rumen microbial metagenome of 3W and 6W calves, but not in 1W calves, suggests the activation of methanogenesis process in calf rumen after the introduction of a solid diet. A recent study has reported that lambs fed only milk replacer and cream produced 84% less methane than lambs fed hay [23]. Moreover, the production of methane increased by 15.9-fold within 4 days of introducing hay to these milk replacer- and cream-fed lambs [23]. Therefore, these observations suggest that the introduction of a solid diet to pre-ruminants may activate the methanogenesis to effectively decrease the H_2_ pressure in the rumen with increasing microbial fermentation. The composition of archaea and the production of methane in lambs have already been manipulated in the long term via manipulating pre-weaned diet [24, 25]. The high heterogeneity and low richness observed in the present study represent an establishing and unstable archaeal community in the pre-weaned calves, which can easily be altered via diet. Thus, the alteration of rumen methanogens during early life through pre-weaned calf feeding strategies can be used to enhance microbial fermentation and to decrease methanogenesis in the rumen.

The use of microbial metagenomics together with DNA- and RNA-based quantification in the present study revealed an absence of methanogenic archaea and protozoa in the rumen of calves at birth. While past culture-based studies [26, 27] reported that archaea colonization began 2-4 days after birth, Guzman and colleagues [28] detected archaea in the rumen samples collected within 0–20 min after birth using qPCR-based approach. Similar to archaea, protozoa were not detected in the rumen of newborn calves (0-day) used in the present study. Currently, protozoa colonization has only been studied using culture-based approaches [29, 30] that report the establishment of ciliate protozoa in the rumen that required a well-establish bacterial community. Thus, well-designed future studies combining both culture-dependant and high-throughput techniques are necessary for in-depth understanding of the initial colonization of rumen archaea and protozoa.

**Fig. 5:**
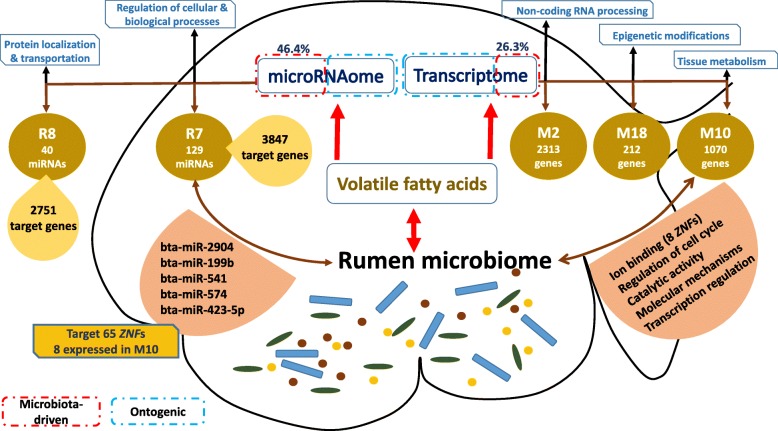
Proposed host-microbial interactions and their regulatory mechanisms in the developing rumen. Early rumen microbiota alters the rumen development via direct and indirect (miRNAs) interactions with the transcriptome. Microbial-derived VFAs are associated with genes involved in ruminal tissue metabolism (M10 gene module), non-coding RNA processing (M2 gene module), and epigenetic modifications (M18 gene module) as well as miRNAs regulating epithelial morphogenesis (R7 miRNA module). miRNAs regulate the host transcriptome either in response to microbial metabolites/rumen microbiota or directly during the early rumen development

RNA-seq-based profiling of host transcriptome has widely been studied in cattle to understand the changes occurring in the rumen tissue with weaning, age, diet, and metabolic disorders at the molecular level of the system biology [9, 31]. The present study explores the postnatal changes in the host transcriptome and the molecular mechanisms behind host-microbial interactions during the rumen development process. Integrated analysis of the host transcriptome and the microbial metagenome revealed the potential molecular mechanisms behind early rumen development, which could be divided into microbial-driven and ontogenic mechanisms (Fig. 5). Only 3 host gene modules (3595 genes, 26.3% of transcriptome) and 2 host miRNA modules (169 miRNAs, 46.4% of microRNAome) were positively or negatively associated with the concentration of VFAs and the development of papillae, indicating that only a portion of host transcriptome was microbial-driven, while majority of them were ontogenic (Fig. 5). Sommer and colleagues [32] have also reported that 10% of the intestinal transcriptome of adult mice is regulated by intestinal microbiota. Our findings, however, suggest more intensive microbial-driven regulation of neonatal rumen tissue transcriptome. The ontogenic miRNA and gene modules revealed 3 miRNA-mRNA pairs (miR-25 and fatty acid-binding protein 7 (*FABP7*); miR-30 and integrin-linked kinase (*ILK*); miR29a and platelet-derived growth factor α polypeptide (*PDGFa*)) involved in the rumen development (Fig. 5). *FABP7* is involved in “fatty acid uptake, transport, and metabolism” [33] and *ILK*-mediated signal transduction in “cytoskeletal organization” [34], and *PDGFa* is involved in intestinal villus morphogenesis [35]. The ontogenic control of the calf rumen development has been suggested previously [36]; however, the present study mainly focuses on the microbial-driven molecular mechanisms, as they are the black box of rumen development.

The identified host genes in the M10 gene module and predicted target genes of the R7 miRNA module provided a common ground to identify host-microbial interactions and their potential regulatory mechanisms in the developing rumen (Fig. 5). Approximately 22% of host genes co-expressed in the M10 gene module (235/1070) were similar to the differentially expressed genes identified in a previous study examining the rumen epithelial gene expression changes when calves were weaned from milk replacer (42 days) to hay/grain (56–72 days) [9]. These 235 common genes were differentially expressed in rumen epithelial transcriptome, when calves were weaned from a milk replacer-based diet (42 days) to hay/grain-based diet (56–72 days), but not with calf age while they received milk replacer from days 14 to 42 [9]. In the present study, 87 out of these 235 genes were differentially expressed, when week 1 was compared against weeks 3 and 6, after the introduction of a solid diet. The strong positive correlations between these host genes and the concentration of VFAs suggest that they may be responsive to diet-driven changes in the rumen fermentation and may facilitate the early rumen development. Connor and colleagues [9] also identified peroxisome proliferator-activated receptor-α (*PPARA*) as an important molecular mechanism of the rumen epithelial development during the weaning process. Although *PPARA* was expressed in all the pre-weaned calves used in this study, it did not display a temporal expression pattern with calf age. However, the expression of *PPARG*, which co-expressed in the M10 host gene module and was correlated with the relative abundance of level 2 microbial functions related to “microbial carbohydrate metabolism,” was upregulated with the calf age. Similar to adult cattle [37], the expression of *PPARG* in the calf rumen tissue was higher than the expression of *PPARA*. *PPARG* is widely studied in ruminants, and its expression level in the rumen is only second to its expression in the bovine adipose tissue [37]. It induces epithelial cell proliferation in the colon [38], upregulates the barrier functions within nasal epithelial cells [39], and is also one of the regulators of intestinal inflammation [40] stimulated via butyrate [41]. Butyrate has been shown to upregulate *PPARG* epigenetically via the inhibition of *HDAC* [42]. The observed negative correlations among the expression of *HDAC3* (co-expressed in the M18 host gene module) and the rumen papillae length and width and the butyrate concentration further reinforces the positive impact of butyrate on early rumen development through the modulation of host transcriptome. A recent study has also reported that gut microbiota-derived butyrate affects histone crotonylation by influencing the expression of *HDAC*s in the intestinal epithelium of mouse [43]. These findings together imply that inhibition of *HDAC*s may be one of the mechanisms of host transcriptome regulation by microbiota and its metabolites (butyrate). Therefore, we speculate that in addition to influencing cell apoptosis [44], butyrate may also be involved in rumen development as a *HDAC* inhibitor and a *PPARG* activator. The observed positive associations between the expression of host *PPARG* and the concentration of VFAs as well as microbial functions related to “microbial carbohydrate metabolism” suggest its involvement in the overall rumen tissue development in response to microbial fermentation.

*ZNF*s are host transcriptional factors that regulate a wide array of functions, including “recognition of DNA,” “packaging of RNA,” “activation of transcription,” “protein folding and assembly,” and “regulation of apoptosis” [45]. The absorption of zinc, a major component of *ZNF*s, also plays an important role in the early rumen papillae development and keratinization in goat kids [46]. The present study revealed that five R7 miRNAs and eight M10 genes related to *ZNFs* were correlated with the abundance of the same bacterial genera (*Prevotella*, *Bacteroides*, *Propionibacterium*, *Ruminococcus*) identified in the rumen microbial metagenomes, suggesting that early microbiota may influence rumen development through zinc absorption, and this interaction may be regulated via miRNAs (Fig. 5). The supplementation of cattle diets with zinc has long been studied to understand its impact on milk production and calf health [47]; however, its role in the early rumen development and the microbial modulation of this process are yet to be understood.

Direct (abundance of bacteria) and indirect (concentration of VFAs) associations between the expression of miRNAs and the early microbiota were evident in this study. A higher proportion of miRNAs (169/364 or 46.4% of microRNAome) than protein-coding genes of a host (3595/13,676 or 26.3% of transcriptome) was associated with the concentration of VFAs, further corroborating our previous findings and speculations on the interactions between miRNAs and microbes [16]. A VFA-associated miRNA from R7, miR-375, inhibits the alveolar epithelial cell differentiation via the Wnt/β-catenin pathway, which participates in “tissue differentiation” and “organogenesis” in rats [48]. The temporal downregulation of miR-375 and its negative associations with the concentration of VFAs and the development of papillae indicate one of the miRNA regulatory mechanisms that can be initiated by microbial metabolites. Thus, the M10 and R7 modules identified from the host transcriptome are indeed biologically important during rumen development and may serve as potential candidates to explore the host-microbial interactions and their regulatory mechanisms (Fig. 5).

In the present study, data generated from the rumen content-associated microbiome was mainly used to explore the host-microbial interactions influencing early rumen development. In addition, we also explored the relationship between the epimural bacterial composition obtained through amplicon sequencing of 16S rRNA and M10 genes or selected GO terms (data not shown). Although the epimural (rumen-tissue attached) microbiota accounts a small proportion of overall rumen microbiome (1–2%), its composition and function may also contribute to tissue development due to its direct interaction with the host. However, no strong associations between the relative abundance of the epimural bacteria taxa and the transcriptome were observed due to the limited number of calves (*n* = 3) used. Future studies to perform metatranscriptomics to sequence both host and the epimural microbiome may be of great importance to completely understand the role of rumen epimural microbiome on early rumen development.

## Conclusions

We demonstrated that rumen colonization began during the birthing process and the pre-ruminant rumen microbiota was highly active and ready to ferment a solid diet even from the first week of life. The VFAs produced by the early microbiome were associated with the rumen tissue metabolism and the development of the epithelium via interacting with the host transcriptome and microRNAome (Fig. 5). We, therefore, propose that early feeding management has a similar importance to the weaning period and may enhance the rumen development and facilitate weaning transition. Our results further indicate that miRNAs may coordinate host-microbial interactions during early rumen development in neonatal calves and this phenomenon may be applicable to early gut development of all mammalian species. Therefore, this study urges in-depth understanding of host-microbial interactions in the developing intestine of neonates to elucidate long-term impacts of early microbiota on the host.

## Materials and methods

### Animal experiments and sampling

All the experimental protocols were approved by the Livestock Care Committee of the University of Alberta (AUP00001012) and were conducted following the guidelines of the Canadian Council on Animal Care. Holstein bull calves at day 0 (*n* = 6, within 5 min after birth), week 1 (1W, *n* = 6), week 3 (3W, *n* = 6), and week 6 (6W, *n* = 6) were obtained from the Dairy Research and Technology Center, University of Alberta (Edmonton, Alberta). Dams with male fetuses were transferred into calving pens a week before the predicted due dates and closely monitored by camera. Newborn calves (*n* = 6) were removed from the dams soon after birth, transferred to a surgery room immediately, and humanely euthanized within few minutes. The whole rumen of each of these newborn calves was collected as a closed section to avoid environmental contamination. The remaining calves (*n* = 18) used in the study were also removed from the dams soon after birth and fed with 2 L of colostrum within 1 h. Calves were fed with 4 L of colostrum/day during the first 3 days postpartum, followed by 4 L of whole milk/day from the fourth day onward throughout the experimental period. From the second week onward, the calves were supplemented with 23% accelerated calf starter (23.0% crude protein, 4.0% crude fat, 9.0% crude fiber, Wetaskiwin Co-op. Association, Wetaskiwin, Alberta, Canada) ad libitum along with 4 L of milk/day. The rumen samples (tissue and content separately) were collected from the pre-weaned calves at week 1, week 3, and week 6 within 30 min after euthanization. Tissue (~ 10 cm^2^) and content (30 ml) samples of older calves were collected at the bottom of the ventral sac, and the site of sampling kept constant for all the animals. All samples were snap-frozen in liquid nitrogen and stored at − 80 °C.

### Analysis of the rumen microbiome

#### Profiling content-associated microbiome using whole genome-based microbial shotgun metagenomics

**Fig. 6:**
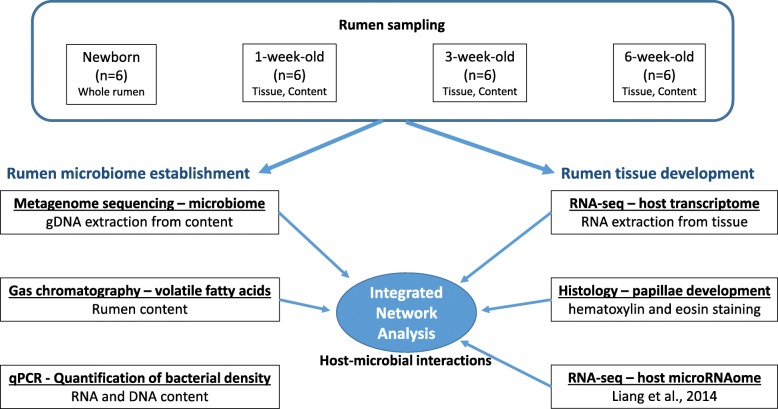
Flow chart depicting the rumen sampling process and approaches used to derive host-microbial interactions of the neonatal rumen

Total DNA was extracted from the rumen content sample using the repeated bead-beating plus column method [49]. Due to the lack of contents, DNA extraction was performed for tissue and contents together for day 0 calves. DNA libraries (Fig. 6) were prepared for whole-genome sequencing using the Truseq DNA PCR-free Library Preparation Kit (Illumina, CA, USA) following the manufacturer’s instructions. Briefly, the genomic DNA was first normalized with a resuspension buffer to a final volume of 55 μL at 20 ng/μL. Then, 50 μL of the buffer containing genomic DNA was transferred into a Covaris microTUBE (Covaris Inc., MA, USA) for fragmentation using a Covaris S2 focused-ultrasonicator (Covaris Inc., MA, USA). The cleaned-up fragmented DNA was then subjected to end repair and size selection, followed by the adenylation of the 3′ ends and ligation of the adaptor index. Each metagenomic library was quantified using a Qubit 2.0 Fluorometer (ThermoFisher Scientific, MA, USA), and sequencing was performed at Génome Québec (Montréal, Canada) using the HiSeq 2000 system (Illumina, CA, USA).

The demultiplexed (CASAVA version 1.8, Illumina) 100-bp paired-end reads (82.9 Gb) were uploaded into the MG-RAST metagenomic analysis server, version 3.3.9, and paired ends were joined for each sample before submitting for processing [50]. Artificial replicates, host (bovine) DNA, and low-quality (Phred score < 25) sequences were removed from the raw data, and the remaining good-quality sequences were used to assign the taxonomy and functions. All microbial metagenome sequence data were deposited at NCBI Sequence Read Archive (SRA) under the accession number SRP097207 (https://www.ncbi.nlm.nih.gov/sra/?term=SRP097207).

The taxonomic abundance was analyzed using the best-hit classification method and the M5NR annotation source within the MG-RAST platform. The functional abundance of the rumen microbiome was analyzed using the hierarchical classification and the subsystems annotation source in the SEED hierarchy. A maximum cutoff *e* value of 1e−10, a maximum identity of 70%, and a maximum alignment length of 50 were used as data selection criteria for both the taxonomy and function abundance analyses. The taxonomic and functional abundances were then subjected to pairwise comparisons (0-day vs. 1-week; 1-week vs. 3-week; 1-week vs. 6-week; 3-week vs. 6-week) using metastats [51] to explore the rumen microbiome changes throughout calf growth. Multiple test correction was performed using Benjamini and Hochberg [52], and significant comparisons were declared at FDR < 0.05.

### Estimation of bacterial/archaeal density using quantitative real-time PCR

DNA- and RNA-based quantitative real-time PCR (Fig. 6) was performed to estimate the bacterial (total bacteria, *Ruminococcus flavefaciens*, *R. albus*, *Eubacterium ruminantium*, *Prevotella ruminicola*) and total archaeal density using SYBR green chemistry (Fast SYBR® Green Master Mix, Applied Biosystems) with the StepOnePlus real-time PCR system (Applied Biosystems, Foster City, CA, USA) and group-specific primers (Table 5.1). The bacterial densities were calculated using the equation described by Li et al. [53].

### Measurement of rumen papillae and volatile fatty acids

Rumen tissue sections (~ 1 cm^2^) adjacent to the sample collected for RNA and DNA extraction were collected into cassettes and then fixed in 10% formalin. After 24 h of fixing in formalin, the cassettes were stored in 70% ethanol until further processing. The rumen tissue samples were embedded in paraffin blocks, and 4–5-μm sections were stained with hematoxylin and eosin at Li Ka Shing Centre for Health Research Innovation (Edmonton, Alberta, Canada). The height and width of the rumen papillae (20 papillae/calf; Fig. 6) were measured using the Axiovision software (Zeiss, Oberkochen, Germany).

Concentration of ruminal VFAs (Fig. 6) was quantified using a Varian 430-gas chromatograph (Varian, Walnut Creek, CA) with a Stabilax®-DA column (Restek Corp., Bellefonte, PA). The concentrations of acetate, propionate, butyrate, isobutyrate, valerate, and isovalerate were calculated according to the method described in Guan et al. [54].

### Transcriptome profiling and integration with rumen microbiome and calf phenotypic traits

#### Profiling rumen transcriptome using RNA-seq

Total RNA was extracted from the rumen tissue samples (Fig. 6) using the mirVana™ miRNA Isolation Kit (Ambion, CA, USA), and libraries were prepared for RNA-seq using the TrueSeq RNA Sample Preparation Kit v2 (Illumina, CA, USA) to enrich poly-A tailed host mRNA with oligodT beads. RNA libraries were sequenced at Génome Québec (Montréal, Canada) using the HiSeq 2000 system (Illumina, CA, USA) to obtain 100-bp paired-end reads. Demultiplexed reads (CASAVA version 1.8, Illumina) were aligned to the bovine genome (UMD 3.1) using Tophat 2.0.10 with the default parameters [55], and only the reads mapped to bovine genome were used for further analysis. The number of reads/gene was determined by using output files from TopHat2 alignment (mapping file) and ENSEMBL bovine gene annotation (GTF file, v75.30, http://uswest.ensembl.org/) with htseq-count (http://www-huber.embl.de/users/anders/HTSeq/). The expression levels of host genes were calculated by normalizing the reads number to counts per million (CPM) reads using the following equation: CPM = (reads number of a gene/total mapped reads number per library) × 1,000,000.

The differentially expressed (DE) host genes between two adjacent age groups (0-day vs. 1-week, 1-week vs. 3-week, and 3-week vs. 6-week) and between 1-week and 6-week were identified using bioinformatics tool edgeR [56]. Only the high abundance host genes (CPM > 5 in at least 50% of the samples) were subjected to DE analysis, and the fold change (FC) was defined as the ratio of arithmetic means of CPM between the two comparison groups. The significantly DE host genes were declared using false discovery rate (FDR < 0.05) obtained a multiple test correction approach [52] and FC > 1.5. Sequencing data were deposited in the publicly available NCBI GEO database and are accessible through GEO series accession number GSE74329 (https://www.ncbi.nlm.nih.gov/geo/query/acc.cgi?acc=GSE74329).

All Gene Ontology (GO) terms and Kyoto Encyclopedia of Genes and Genomes (KEGG) pathways enrichment of host genes were performed using Database for Annotation, Visualization and Integrated Discovery (DAVID), http://david.abcc.ncifcrf.gov [57]. All the analyses were performed using the functional annotation clustering option, and the significant GO terms and KEGG pathways were declared at *P* < 0.05 and molecule number > 2. Ingenuity pathway analysis (IPA, Ingenuity Systems, www.ingenuity.com) was used to analyze the top host functions of the rumen tissue and the functions of DE genes with a threshold level of *P* < 0.01 to enrich the significant biological functions. The *z*-score algorithm from IPA and FC were used to predict the increase or decrease expression changes of DE genes (*z* > 2—significantly increased functions; *z* < − 2—significantly decreased function). If there were no significantly increased or decreased functions, the functions with the smallest *P* values were selected.

MicroRNAome data of the same neonatal calves profiled using RNA-seq was obtained from our previously published work Liang et al. [16]. All expressed miRNAs (CPM > 1 in at least one sample) were used to further explore their regulatory mechanisms behind the host-microbial interactions in the developing rumen.

#### Exploring associations between rumen microbiome and rumen tissue transcriptome using network analysis

The interactions among the host protein-coding genes, miRNAs [16], and microbial metagenomes were explored through network analysis and correlation analysis. Weighted gene co-expression network analysis (WGCNA) [58] was performed to understand the link between the host transcriptome/miRNAome (profiles generated from the same calves) and the calf phenotypic traits (calf age, concentration of acetate, propionate, butyrate, valerate, isobutyrate, isovalerate and total VFAs, papillae length and width).

All expressed protein-coding genes (15,139, CPM > 1 in at least 1 sample) in rumen tissue samples collected from all calves (except day 0) and all expressed miRNA (412) in all older calves (1-week, 3-week, and 6-week) were used in WGCNA analysis (R package v3.4.1). First, a gene co-expression network was constructed based on the correlation/co-expression patterns among genes/miRNAs using pickSoftThreshold function. Then, the mRNA/miRNA modules (clusters of densely interconnected genes/miRNAs) were identified using a hierarchical clustering approach. Module detection (blockwiseModules in WGCNA) functions were performed with the following parameters: maxBlockSize of 16,000, minModuleSize of 30, and reassignThreshold of 0. This approach generated 29 mRNA modules (defined as M1-M29) and 9 miRNA modules (defined as R1-R9). The correlation coefficients between the gene/miRNA module and calf phenotypic traits were calculated using the following linear regression equation. *Y*_*i*_ = *β*_0_ + *β*_1_.*X*_*i*_ + *e*_*i*_, where *Y*_*i*_ is the expression level of a module eigengene (module eigengene is defined as the first principal component of a given module and used to represent the overall expression level of a module) in the *i*th sample, *β*_0_ is the random intercept, *β*_1_ is the slope coefficient, *X*_*i*_ is the value of calf phenotypic traits in the *i*th sample, and *e*_*i*_ is the random error.

The associations between the host transcriptome and the rumen bacteria were further explored using the host genes co-expressed in the M10 module of the mRNA network, the miRNAs co-expressed in the R7 module of the miRNA network, and the relative abundance of the identified rumen bacterial genera. The associations between the host transcriptome and the microbial functions were explored using the relative abundance of level 2 microbial functions in the SEED subsystems hierarchy and GO terms enriched under “host carbohydrate metabolism” (GO: 0005975, 20 genes), “tight junction protein genes” (GO: 0005923, 14 genes), “membrane transportation” (GO: 0008643, 14 genes), and “epithelial development” (GO: 0060429, 34 genes).

Target genes of the R7 and R8 miRNA modules were predicted using both TargetScan (http://www.targetscan.org) and mirBase (http://www.mirbase.org/). The target genes predicted by both methods were then compared with the rumen tissue transcriptome generated in the present study to identify the number of target genes expressed in the pre-weaned calf rumen tissue.

### Statistical analysis

The DNA- and RNA-based bacterial/archaeal density, concentration of VFAs, and papillae length and width were analyzed using the mixed procedure in SAS (SAS 9.4, SAS Inc., Cary, NC) and one-way analysis of variance. The following statistical model was fitted to test the effect of calf age on bacterial/archaeal densities, papillae length and width, and the concentration and molar proportion of VFAs: *Y*_*ij*_ = *μ* + *A*_*i*_ + *e*_*ij*_, where *Y* is the bacterial/archaeal density (total bacteria, *R. flavefaciens*, *R. albus*, *E. ruminantium*, *P. ruminicola*, total archaea), VFA concentration/molar proportion, papillae length or width; *μ* is the mean; *A* is the calf age; and *e* is the residual error. The correlations among the concentration of VFAs, bacterial densities, and papillae length and width were identified using PROC CORR in SAS. Differences in LSM were declared at *P* < 0.05 using the PDIFF option in SAS when applicable.

## Additional files

Additional file 1: An excel file containing the relative abundance of rumen bacterial community and differentially abundant microbial functions. (XLSX 27 kb)

Additional file 2: A PDF containing supplementary figure legends, all supplementary tables (Table S1 & Table S2) and supplementary figures (Figure S1 to Figure. S5). (PDF 2107 kb)

Additional file 3: An excel file containing commonly expressed transcripts of the rumen tissue and top 3000 functions identified from the rumen tissue transcriptome. (XLSX 412 kb)

Additional file 4: An excel file containing differentially express transcripts among different age groups and their functions. (XLSX 102 kb)

Additional file 5: An excel file containing miRNAs co-expressed in R7 and R8 miRNA-modules and their predicted target. (XLSX 177 kb)

Additional file 6: Review history. (DOCX 34 kb)

## Data Availability

RNA-seq sequencing data are available at the NCBI Gene Expression Omnibus database under the accession number GSE74329 [59]. All microbial metagenome sequence data are available at the NCBI Sequence Read Archive under the accession number SRP097207 [60]. MicroRNA data used are available through the GEO Series accession number GSE52193 [61].
