# Supplementary material for: Regulation of rumen development in neonatal ruminants through microbial metagenomes and host transcriptomes
Source: Genome Biol. 2019 Aug 23;20:172. doi: 10.1186/s13059-019-1786-0 (PMC6708143; doi:10.1186/s13059-019-1786-0)
Supplement: Supplementary file 2 — A PDF containing supplementary figure legends, all supplementary tables (Table S1 & Table S2) and supplementary figures (Figure S1 to Figure. S5). (PDF 2107 kb) [file 13059_2019_1786_MOESM2_ESM.pdf]

Regulation of rumen development in neonatal ruminants through microbial metagenomes and host transcriptomes

Nilusha Malmuthuge[a,1], Guanxiang Liang[a,1], Le Luo Guan[a,2]

[a] Department of Agricultural, Food and Nutritional Science, University of Alberta, Edmonton, Alberta, Canada T6G 2P5

[1]These authors contribute equally to this work

[2]Corresponding Author: 4-16F, Agriculture/Forestry Center, Department of Agricultural, Food and Nutritional Science, University of Alberta, Edmonton, Alberta, Canada T6G 2P5. Tel: +1 780 4922480; Fax: +1 780 4924265; E-mail: lguan@ualberta.ca

E-mail addresses

Nilusha Malmuthuge : malmuthu@ualberta.ca

Guanxiang Liang : guanxian@vet.upenn.edu

Le Luo Guan : lguan@ualberta.ca

Supplementary Figure Legends

Fig. S1. Composition of rumen microbiome in calves from birth (at birth) up to six weeks of life. Each data series represent mean relative abundance of microbial group as a % of total detected microbiota.

Fig. S2. Gene modules detected through WGCNA. (A) Number of transcripts detected and percentage of reads belonged in each gene module. B. The most relevant function of gene modules M10, M2, and M18 obtained through GO term enrichment.

Fig. S3. Protein coding genes belong to mitochondrial complex. Red color indicated genes co-expressed in M10 gene module identified via WGCNA.

Fig. S4. Level 2 microbial functions associated with host protein coding genes expression. (A) Level 2 microbial functions associated with ($P < 0.01$, $r^2 \geq 0.98$) host genes involved in rumen tissue carbohydrate metabolism (GO: 0005975, 20 genes). (B) Level 2 microbial functions associated with ($P < 0.01$, $r^2 \geq 0.98$) host genes involved in membrane transportation (GO: 0008643, 14 genes). Right side panel represents genes co-expressed in M10 gene module. FUCA1 – fucosidase alpha L-1; SLC16A1 – solute carrier family 16 member 1; SLC35A3 - solute carrier family 35 member 3.

Fig. S5. Level 2 microbial functions associated with ($P < 0.01$, $r^2 \geq 0.98$) host genes involved in tight junction protein genes (TJs) (GO: 0005923, 14 genes). Upper network represents TJs correlated with microbial carbohydrate metabolism. Lower network represents TJs correlated with other microbial functions. Left side panel represents genes co-expressed in M10 gene module. CLDN23 – claudin 23.

Table S1. Association between rumen papillae development, concentration of VFAs, and cellulolytic bacteria

	**Acetate**	**Propionate**	**Butyrate**	**Valerate**	**Isobutyrate**	**Isovalerate**
Papillae length[1]	0.7***	0.6***	0.7***	0.6**	0.7***	0.6***
Papillae width	0.8***	0.8***	0.7***	0.7***	0.8***	0.7***
R. flavefaciens	0.6***	0.4	0.6***	0.4*	0.4*	0.3

[1] Pearson correlation coefficient.

* $P < 0.1$; ** $P < 0.05$; *** $P < 0.01$.

Table S2. Primers used in qPCR to estimate bacterial/archaeal densities

	**Forward primer (5'-3')**	**Reverse primer (5'-3')**	**Source**
Total bacteria	actcctacgggaggcag	gactaccagggtatctaatcc	Stevenson & Weimer, 2007
E. ruminantium	ctcccgagactgaggaagcttg	gtccatctcacaccaccgga	Stevenson & Weimer, 2007
P. ruminicola	gaaagtcggattaatgctctatgttg	catcctatagcggtaaacctttgg	Stevenson & Weimer, 2007
R. albus	gttaacagagggaagcaaagca	tgcagcctacaatccgaactaa	Stevenson & Weimer, 2007
R. flavefaciens	tggcggacgggtgagtaa	ttaccatccgtttccagaagct	Stevenson & Weimer, 2007
Total archaea	ccggagatggaacctgagac	cggtcttgcccagctcttattc	Zhou et al., 2010

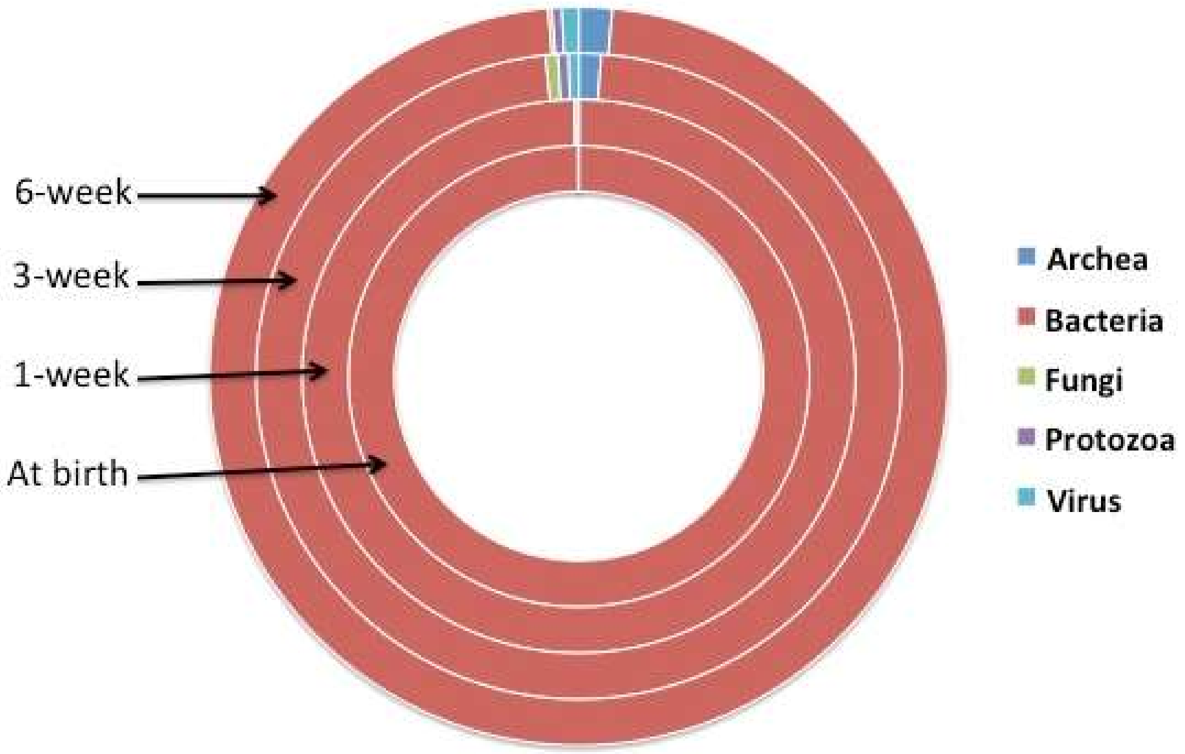

Malmuthuge et al., Fig. S1

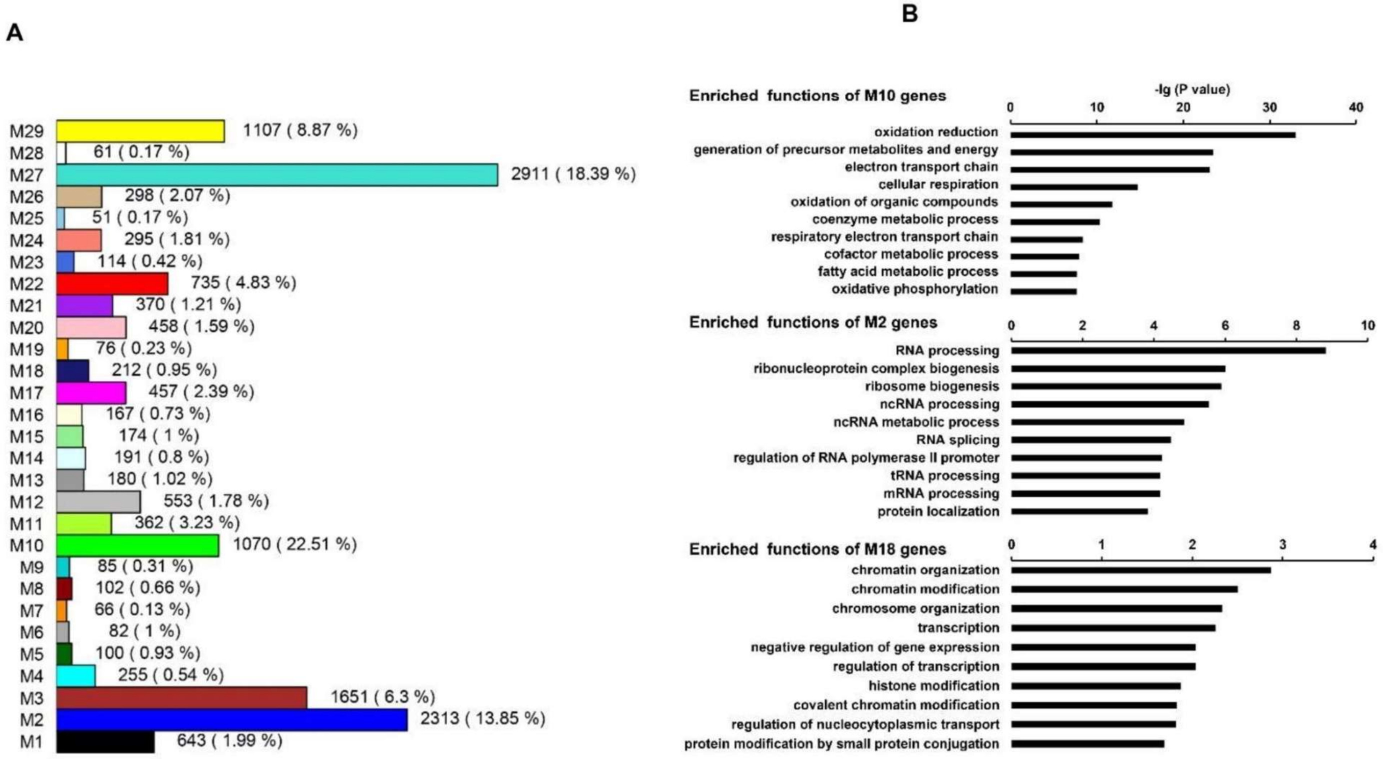

Malmuthuge et al., Fig. S2

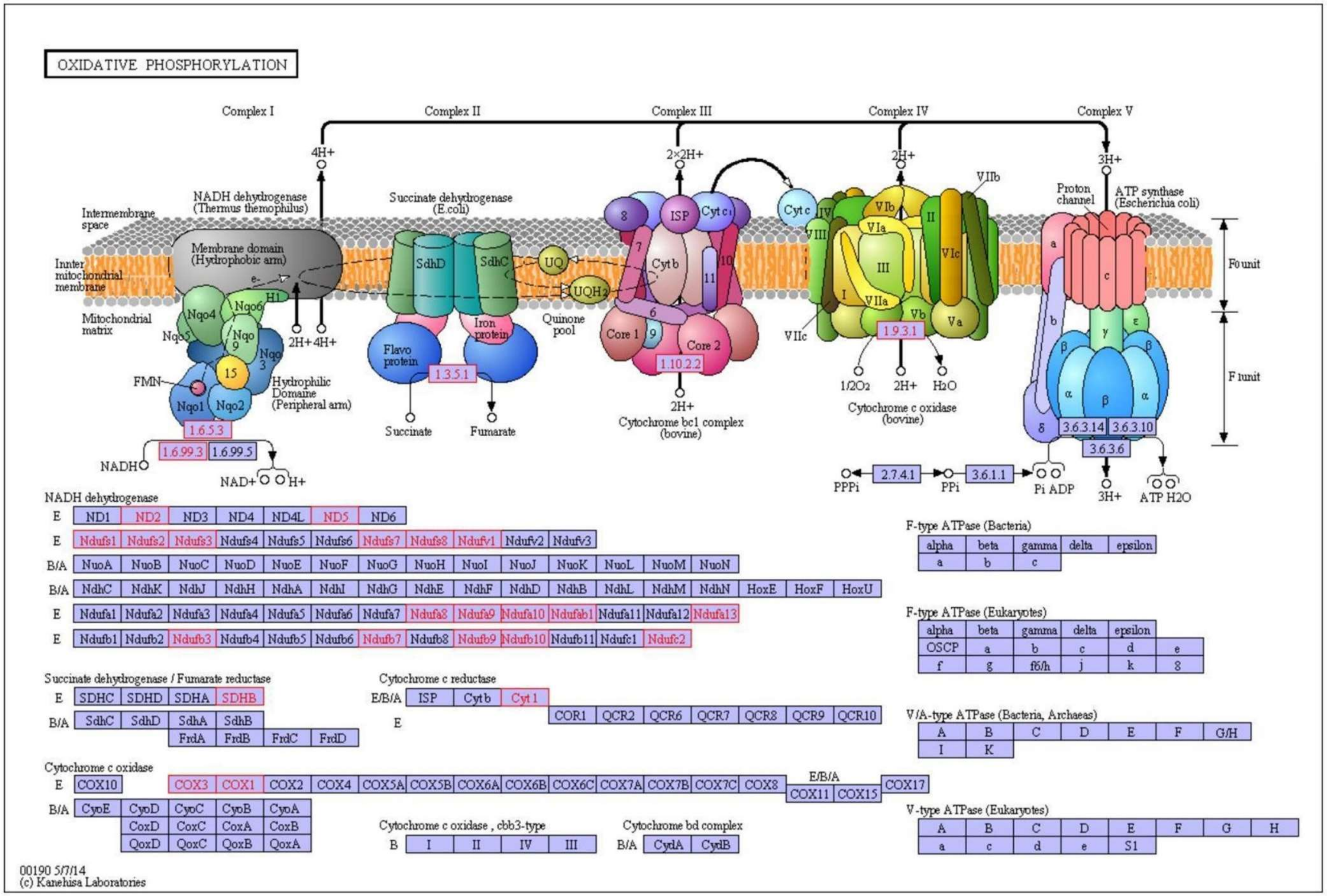

Malmuthuge et al., Fig. S3

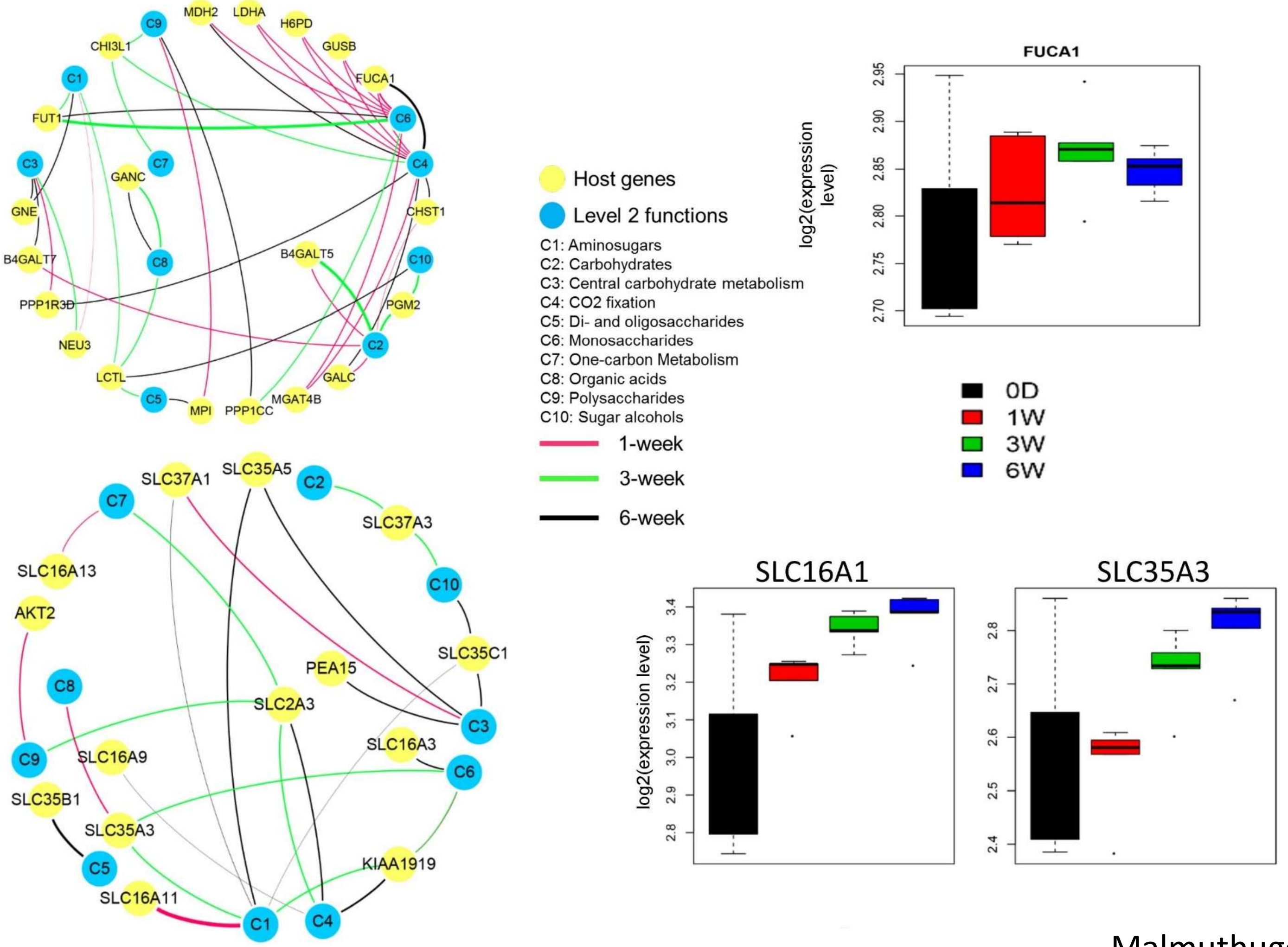

Malmuthuge et al., Fig. S4

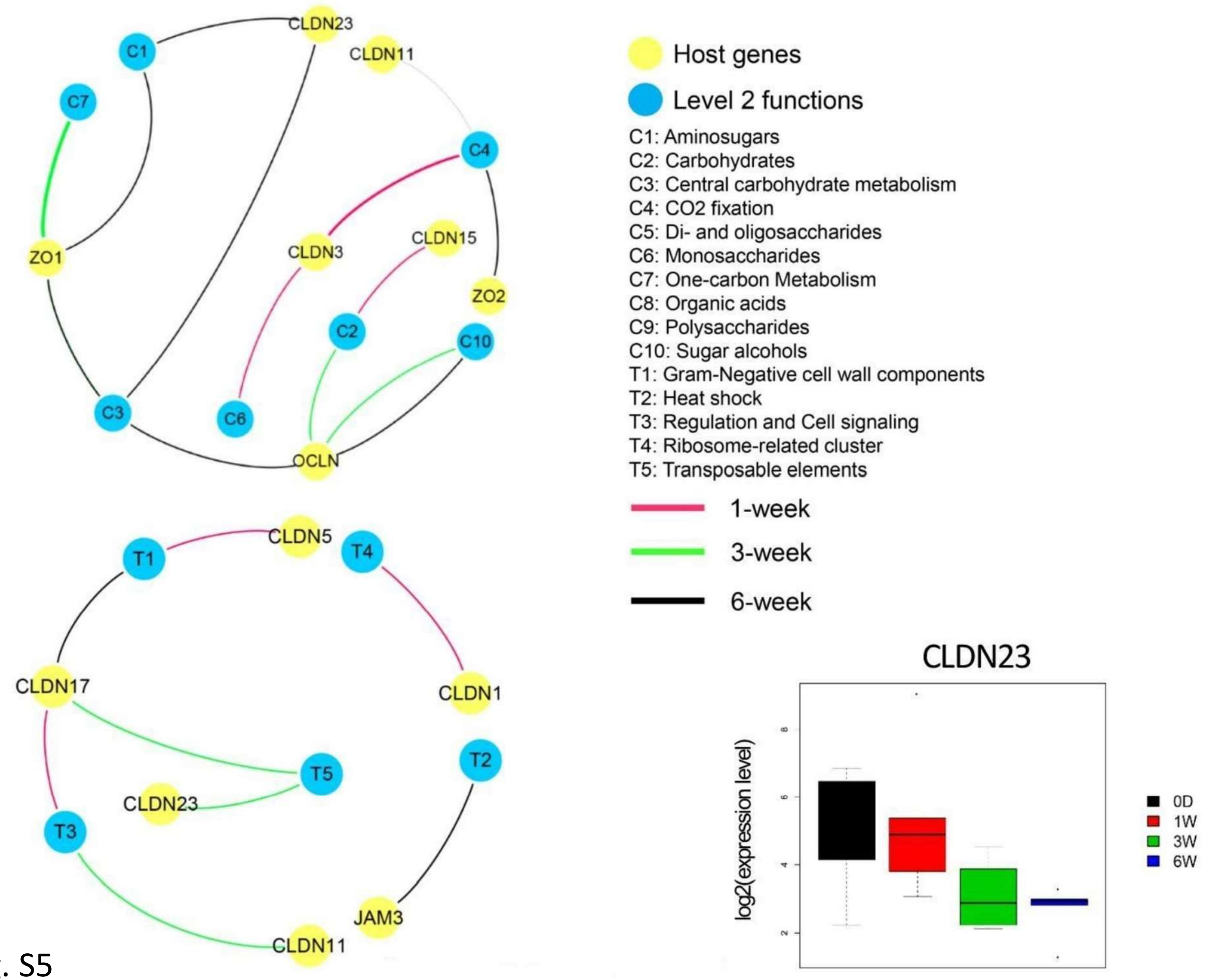

Malmuthuge et al., Fig. S5
