# Supplementary material for: Regulation of rumen development in neonatal ruminants through microbial metagenomes and host transcriptomes
Source: Genome Biol. 2019 Aug 23;20:172. doi: 10.1186/s13059-019-1786-0 (PMC6708143; doi:10.1186/s13059-019-1786-0)
Supplement: Supplementary file 6 — Review history. (DOCX 34 kb) [file 13059_2019_1786_MOESM6_ESM.docx]

Review History

**First round of review**

**Reviewer 1**

**Are you able to assess all statistics in the manuscript, including the appropriateness of statistical tests used?** *Yes, and I have assessed the statistics in my report.*

**Comments to author:**

*1. General comments:*

*The research on the extent that microbiota and host interact to develop the rumen during the first period of life is of high interest for the scientific community. The results are novel and suggest a bacteria-driven transcriptional regulation of the development of the rumen at early life via miRNAs.*

*However, there are some specific comments to be address as follows:*

*2. Specific comments:*

*Line     Comment*

*L33-35     Could this be underestimated because not all microbial metabolites are detected?*

*L34     Because you are analysing both host and microbial genes I suggest to define the genes as host or microbial here and in the entire manuscript.*

*L36     See above comment*

*L104-106     How sure are you that this is not an error of detection that you do not find archaea and protozoa?*

*L107-108     Please could you explain the level 1 and 2 system of SEED for readers who are not familiar with this database?*

*L119     Is this significant?*

*L131-136     Some indication of significance of this increase would be necessary.*

*L202     Are these network modules indicators (M1 to M29) given by you or do they correspond to a specific database? Please clarify it also in the manuscript.*

*L219     Please indicate why did you choose the M10 module?*

*L224     Please indicate what genes these are as described in an earlier comment (L34).*

*L242     Is it right that you explored the rumen epimural bacteria only on 3 calves? If yes, why not on all animals?*

*L249     Are these network modules indicators (R1 to R9) given by you or do they correspond to a specific database? Please clarify it also in the manuscript.*

*L336     Please rewrite miRNA-mRNA to be consistent with the description.*

*L273-405     The discussion of the results is excellent.*

*L439-440     Please could you described the site of sampling more defined and how the samples were taken, what amount, and how stored, etc.*

*Figure 1 B     Please improve in the figure labels the writing of the phyla*

*Figure 1 D     Please improve the axis description on the ordinate*

*Figure 1 E     Please improve in the figure labels the writing of the families*

*Figure 2 B     I guess the Gene modules subfigure is Fig 2 (B), please define subfigure.*

*Figure 3 B     The abscissa has no axis description and the different colours are not defined.*

**Reviewer 2**

**Are you able to assess all statistics in the manuscript, including the appropriateness of statistical tests used?** *Yes, and I have assessed the statistics in my report.*

**Comments to author:**

*General comments:*

*This is an excellent paper which adds substantially to the understanding in this area. However, the paper lacks clarity at the minute and requires some attention to improve readability before publication.*

*Specific Comments:*

*Abstract*

*Lines 19-21: needs better explanation for those who may read the article from a non-rumen background.*

*Line 22: including ‘rumen microbial shotgun metagenomics’ and ‘host’ microRNAome.*

*Line 23: ‘investigate the extent and functional basis that rumen ..’*

*Background needs some information on the animal experimental design.*

*Results*

*Line 26: Active bacteriome?  You don’t mention anything about RNA microbial analysis in the background section of the abstract.*

*Lines 27-29: This doesn’t make much sense, what do you mean by adult-like microbiota?*

*Line 34: insert ‘host’ transcriptome as easy for reader to mix between which datasets are discussed.*

*Line 35: metabolites used but you looked at gene level only, not on a metabolite level?*

*Line 36: ‘a gene module consisting of 1070 genes was enriched in association with VFAs  (need to noted which VFAs), papillae…’ Also be clear what you mean by associated did the gene abundances go up or down?*

*Line 40: ‘with target genes involved in…’*

*Introduction*

*Line 70: You don’t explain the 4 compartments of stomach which define a ruminant before mentioning the monogastric system.*

*Line 72: Need better word than ‘proper’*

*Line 77: pre-ruminant terminology doesn’t seem right.*

*Lines 84-85:  My main challenge with this paper is the lack of clarity over the techniques that were used n their totality.  The techniques defined in materials and methods do not match to the aims or abstract.*

*Materials and methods*

*This section needs some improvement. It seems you also completed metataxonomical analysis and qPCR but this isn’t mentioned in the abstracts or aims section of introduction.*

*In general the materials and methods section needs a diagram outlining the sampling and analysis conducted as it’s very confusing throughout the paper.*

*Transcriptome profiling. Suggest separate miRNA section from total transcriptome section so it makes understanding easier.  Also I’m confused, would the total metagenomic sequencing not have revealed sequences for non-bacterial microbes? Also for the tissue RNA sequences would this not have contained microbial metatarnscriptome sequences? Need to increase clarity running throughout paper.*

*Results*

*Lines 138-142: I could only see Phylum level data in the supplementary data?*

*Line 170: metagenomic not metagenomics*

*Line 180: This is Figure 1G not 1E, but there doesn’t seem to be a 1F? Need to check figure numbering. Also, there are no apparent microbes in the papillae images?*

*Discussion*

*Line 323: what do you mean specifically when you talk about rumen transcriptome? Host? Microbial?*

*Don’t refer to figures/tables in discussion, discuss data in a more holistic manner.*

*Conclusions*

*Remove Figure 5.*

**Authors Response**

**Point-by-point responses to the reviewers’ comments:**

Reviewer #1: 1. General comments:

The research on the extent that microbiota and host interact to develop the rumen during the first period of life is of high interest for the scientific community. The results are novel and suggest a bacteria-driven transcriptional regulation of the development of the rumen at early life via miRNAs.

**Reply:** Thank you for reviewer’s positive comments. We have carefully revised the manuscript to address all the concerns raised.

However, there are some specific comments to be address as follows:

2. Specific comments:

Line    Comment

L33-35  Could this be underestimated because not all microbial metabolites are detected?

**Reply:** The authors agree with the reviewer that this statement is not specific. Abstract and results have been revised to indicate that only the volatile fatty acids were measured in the present study. Line 24-25 “quantification of volatile fatty acids (VFAs - acetate, propionate, butyrate, total)”; line 34 “associated with VFAs”

L34     Because you are analysing both host and microbial genes I suggest to define the genes as host or microbial here and in the entire manuscript.

L36     See above comment

**Reply:**  The manuscript has been modified based on reviewer’s suggestions. We used host genes (for host protein coding genes) and microbial functions (for microbial genome) through the manuscript to avoid confusions. eg: Line 31 “one host gene module (M10; “tissue metabolism”) was positively, while two other host gene modules”; Line 34 “Eight M10 host genes”.

L104-106        How sure are you that this is not an error of detection that you do not find archaea and protozoa?

**Reply:** We appreciate your comments on this regard. Past studies have indicated that archaeal methanogens and protozoa only colonize rumen after a few days of life due to their origin of environment and preference for nutrients (Stewart et al., 1988; Morvan et al., 1994). These studies further report that it is necessary to provide suitable ecological niches (sources of energy, carbon, nitrogen minerals and trace nutrients for growth, at ambient pH) for their colonization, thus these microbial groups start to colonize after such conditions are established by the pioneer bacteria. Although a recent paper reporting the presence of archaeal species (qPCR-based) in the rumen of newborn calves within the first 72 hours (Guzman et al., 2015.), it was based on DNA and it is unclear whether the true values are within qPCR detection limit because the authors presented the abundance (Abundance, as Log10 copy number per μl of 16S ribosomal DNA, was expressed relative to the lowest value among all tested species) data relative to the lowest value obtained from qPCR. While this is the only study to report the presence of archaea at birth, presented data format is also rather different from the general qPCR format that prevent us from evaluating the presence of methanogens in the whole GIT of calves at birth.

In the present study, we used both DNA-based and RNA-based qPCR based quantification of same day 0 samples as a validation approach for microbial metagenomes sequencing results (no methanogen and protozoal genes were detected). Our quantification cycles (Cq) from newborn samples were similar to the no template control (>35 cycles) at both DNA and RNA levels. Therefore, in the future, it is necessary to validate whether the reported detection of methanogens in the gut of newborn calves is true and whether they are originated from the initial exposure to dam’s microbiota or the natural colonization process in newborn calves using the culture-based methods, and /or with more controlled experimental designs.

Similar to archaea, protozoa have also been shown to establish later in life (Eadie, 1962; Fonty et al., 1988.). Further, Fonty et al., (1988) reported the establishment of the ciliate protozoa in the rumen requires a well-established bacterial community. However, there has been no culture independent based study yet to explore the protozoa community at birth. A brief discussion on the establishment of archaea and protozoa was added to revised manuscript (Line 332-342).

*References:*

Eadie, 1962. The development of rumen microbial populations in lambs and calves under various conditions of management. J. Gen. Microbiol. 29: 579-588;

Fonty et al., 1988. Establishment of ciliate protozoa in the rumen of conventional and conventionalized lambs: influence of diet and management conditions. Can. J. Microbiol. 34:235-241

Stewart et al., (1988). The establishment of rumen microbial communities. Anim. Feed Sci. Technol. 21 69–97. 10.1016/0377-8401(88)90093-4;

Morvan et al., (1994). Establishment of hydrogen-utilizing bacteria in the rumen of the newborn lamb. FEMS Microbiol. Lett. 117 249–256. 10.1111/j.1574-6968.1994.tb06775.x

Guzman et al., 2015. Presence of Selected Methanogens, Fibrolytic Bacteria, and Proteobacteria in the Gastrointestinal Tract of Neonatal Dairy Calves from Birth to 72 Hours. PLoS ONE 10(7): e0133048. <https://doi.org/10.1371/journal.pone.0133048>

L107-108        Please could you explain the level 1 and 2 system of SEED for readers who are not familiar with this database?

**Reply:**  Thank you for reviewer’s valuable comment. We modified the manuscript to include an explanation of different levels presented in the manuscript at the first use of terms. Please see,  Lines 106-108 “(subsystems hierarchy - collection of related functional roles represented in four level hierarchy)”; “(level 1 - highest level of subsystem, eg: protein metabolism)”; “level 2 (sub pathways within a major metabolic pathway eg: protein biosynthesis)”; “microbial genes (level 4)”

L119    Is this significant?

**Reply:** Yes, statistical differences were indicated in Fig. 1A. In the revised version we have also included statistical significances in the text.

L131-136        Some indication of significance of this increase would be necessary.

**Reply:** Yes, statistical differences were indicated in Table1. In the revised version we have also included statistical significances in the text other than as figure legends/table footnotes.

L202    Are these network modules indicators (M1 to M29) given by you or do they correspond to a specific database? Please clarify it also in the manuscript.

**Reply:** Thank you for reviewer’s comment that helps us to realize the importance of adding a summary of methods used. Originally, weighted gene co-expression network analysis (WGCNA) package assigned indicators to detect modules using its standard colour codes (eg: original indicator of M10 is MEgreen, original indicator of M2 is MEblue). The original colour codes can be found in supplementary figure 2A. Later, we assigned M1-M29 indicators for these colour code based original module indicators to simplify data presentation. The manuscript has been modified based on reviewer’s comments to add detailed procedure of WGCNA used to generate gene modules. Line 204 “(defined as M1-M29 modules; Figure 2B, Supplementary Figure 2)”. We have also added a new paragraph (Lines 584-598) in materials and methods section to provide details on WGCNA, hoping that it could increase the clarity of methods used and data presented.

L219    Please indicate why did you choose the M10 module?

L224    Please indicate what genes these are as described in an earlier comment (L34).

**Reply:** Among the detected gene modules, although there were a total of 1070 co-expressed genes (third highest number of genes co-expressed in all detected modules), expression of genes belonged to M10 module accounted for 22.5% of total sequencing reads, making M10 the largest gene module detected depend on gene expression level. Moreover, the enrichment of module functions revealed that genes co-expressed in M10 module are related to rumen tissue metabolism. Further, M10 is the only positively correlated gene module with rumen papillae length and width, concentration of all VFAs including total VFA, and calf age. The manuscript has been modified based on reviewer’s comments to indicate reasons behind choosing M10 for further analysis. Please see Lines 223-226 “M10 module, which clustered host genes related to rumen tissue metabolism and positively correlated with the concentration of VFAs …….”

L242    Is it right that you explored the rumen epimural bacteria only on 3 calves? If yes, why not on all animals?

**Reply:** Yes, we only used 3 calves for rumen epimural bacteria analysis using 16S rRNA gene amplicon sequencing. In the present study, the main objective was to explore rumen microbiome during the pre-weaning period and its interaction with the host at cellular level of the system biology. Use of whole genome sequencing was not effective to assess tissue attached (epimural) microbiome due to the presence of higher levels of host genomic DNA and thus, we only explored the rumen content microbiome. Yet, we wanted to generate knowledge on early colonization process on rumen tissue-attached community (which is different from the content bacterial community) to provide a complete picture of rumen colonization without shifting our main focus. In our previous studies we have profiled the rumen bacterial communities in both rumen content and tissue of pre- and post-weaned calves (Malmuthuge et al., 2012; Malmuthuge et al., 2014). These studies revealed that tissue attached bacterial community (composition and diversity) is differed from content-associated community. We speculated that the epimural microbiome may have more direct interaction with host. However, we only have limited data (16S amplicon sequencing data of 3 animals), no relationship was identified between the relative abundance of detected taxa and rumen transcriptome. After considering reviewer’s comment further, we have decided to remove this section from the revised manuscript as we thought it might deter reader’s focus. However, in the discussion we addressed the importance of studying the epimural microbiome to fully understand the role of rumen microbiome on early development of rumen (Line 431-441).

*References:*

Malmuthuge et al., (2012). Distinct commensal bacteria associated with ingesta and mucosal epithelium in the gastrointestinal tracts of calves and chickens. FEMS Microbiol Ecol. 79(2):337-47. doi: 10.1111/j.1574-6941.2011.01220.x.

Malmuthuge et al., (2014). Taxonomic identification of commensal bacteria associated with the mucosa and digesta throughout the gastrointestinal tracts of preweaned calves. Appl Environ Microbiol. 80(6):2021-8. doi: 10.1128/AEM.03864-13.

L249    Are these network modules indicators (R1 to R9) given by you or do they correspond to a specific database? Please clarify it also in the manuscript.

**Reply:** Thank you for reviewer’s comment that helps us to realize the importance of adding a summary of methods used. Originally, weighted gene co-expression network analysis (WGCNA) package assigned indicators to detected modules using its standard colour codes (eg: original indicator of R7 is MEblack, original indicator of R8 is MEblue). The original colour codes can be found in supplementary figure 2A. Later, we assigned R1-R29 indicators for these colour code based original module indicators to simplify data presentation and to distinct from mRNA modules. The manuscript has been modified based on reviewer’s comments to add detailed procedure of WGCNA used to generate miRNA modules. Please see Line 256 “(defined as R1-R9 miRNAs modules)” We have also added a new section (Lines 584-598) to materials and methods to provide details on WGCNA, hoping that it will increase the clarity of methods used and data presented.

L336    Please rewrite miRNA-mRNA to be consistent with the description.

**Reply:** The manuscript has been modified based on reviewer’s comments. Please refer to Line 350 in the revised version.

L273-405        The discussion of the results is excellent.

**Reply:** Thank you for reviewer’s positive comment

L439-440        Please could you described the site of sampling more defined and how the samples were taken, what amount, and how stored, etc.

**Reply:** All sampling was done following euthanasia and isolating rumen from the rest of intestine. Samples (tissue and content) were always collected from the bottom of ventral sac. Firstly, we collected content and then after cleaning the tissue with sterile PBS a piece of tissue was collected. The detailed information on the site of sampling and how the samples were taken, what amount, and how stored have been included in the revised version. Please see Lines 475-477 “Tissue (~10 cm^2^) and content (30 ml) samples of older calves were collected at the bottom of ventral sac and the site of sampling kept constant for all the animals. All samples were snap frozen in liquid nitrogen and stored at -80°C.”

Figure 1 B      Please improve in the figure labels the writing of the phyla

Figure 1 D      Please improve the axis description on the ordinate

Figure 1 E      Please improve in the figure labels the writing of the families

Figure 2 B      I guess the Gene modules subfigure is Fig 2 (B), please define subfigure.

Figure 3 B      The abscissa has no axis description and the different colours are not defined.

**Reply:** Many thanks for reviewer’s valuable suggestions, all figures have modified to increase clarity and to improve figure labels. Figure panels were carefully checked to make sure numbering is in order. Axis descriptions were added to figure 3B.

Reviewer #2: General comments:

This is an excellent paper which adds substantially to the understanding in this area. However, the paper lacks clarity at the minute and requires some attention to improve readability before publication.

Specific Comments:

Abstract

Lines 19-21: needs better explanation for those who may read the article from a non-rumen background.

**Reply:** Thank you for reviewer’s comment. We modified the context sentence to provide information for a broad audience (Lines 19-20 “Early rumen development is vital for efficient rumen fermentation that converts plant materials to human edible milk and meat in ruminants.”). We have also revised the abstract according to Genome Biology journal guidelines (limit to 250 words) to only present context and purpose of the study under the background section. Due to the word limitation, we remove methods from the background section. However, in the revised version, before presenting each main result we did our best to let readers know the method used to generate them. For example, Lines 24-26 “Use of microbial metagenomics together with quantification of volatile fatty acids (VFAs - acetate, propionate, butyrate, total) and RNA-based quantitative PCR revealed the colonization of an active bacterial community in the rumen at birth.”

Line 22: including ‘rumen microbial shotgun metagenomics’ and ‘host’ microRNAome.

**Reply:** Thank you for the suggestion to clarify microbial genes from host genes. We have modified the manuscript to distinct microbial functions from host genes throughout the manuscript. We have carefully gone through Marchesi and Ravel, (2015), which defines commonly used terms in microbial analysis using molecular methods. In this paper, authors proposed that “metagenome” is the collection of genomes and genes from the members of a microbiota, whereas “metagenomics” is shotgun sequencing of DNA extracted from an environment sample. We used “microbial metagenomics” for the approach to sequence the rumen microbiome and “transcriptomics (RNA-seq)” for the approaches used to sequence host transcriptome and microRNAome. Eg: Lines 28-29 in the abstract

*Reference:*

Marchesi and Ravel (2015). The vocabulary of microbiome research: a proposal. Microbiome, 3:31. https://doi.org/10.1186/s40168-015-0094-5

Line 23: ‘investigate the extent and functional basis that rumen ..’

Background needs some information on the animal experimental design.

**Reply:** The manuscript has been modified based on reviewer’s comment. Please see Lines 20-21 “Here, we investigated the extent and functional basis of host-microbial interactions regulating the rumen development during the first six weeks of life.”

Results

Line 26: Active bacteriome?  You don’t mention anything about RNA microbial analysis in the background section of the abstract.

Lines 27-29: This doesn’t make much sense, what do you mean by adult-like microbiota?

Line 34: insert ‘host’ transcriptome as easy for reader to mix between which datasets are discussed.

Line 35: metabolites used but you looked at gene level only, not on a metabolite level?

Line 36: ‘a gene module consisting of 1070 genes was enriched in association with VFAs (need to noted which VFAs), papillae…’  Also, be clear what you mean by associated did the gene abundances go up or down?

Line 40: ‘with target genes involved in…’

**Reply:** Following reviewer’s suggestion and the journal’s guideline, we have revised abstract section significantly. First, we shortened the abstract to meet the length limit (250 words), and also following reviewer’s suggestion we included brief statements of methods used to achieve the main results. Specifically, we changed active bacteriome to active bacteria (RNA-based quantification) because this data was generated through RNA-based qPCR. We have also included methods used to generate the data presented in Lines 24-29 (“Use of microbial metagenomics together with quantification of volatile fatty acids (VFAs - acetate, propionate, butyrate, total) and RNA-based quantitative PCR revealed the colonization of an active bacterial community in the rumen at birth. Rumen microbial community later colonized by complex carbohydrate fermenters and archaea with methyl-coenzyme M reductase activity from the first week of life at the absence of a solid diet. Integrated multi-omics approach (microbial metagenomics, host transcriptomics and microRNAomics)”). Again, thanks for the comment to use appropriate terminology to clearly present host transcriptome and microbiome data. We have used “host genes” when presenting transcriptome results in the abstract as well through the manuscript.

Introduction

Line 70: You don’t explain the 4 compartments of stomach which define a ruminant before mentioning the monogastric system.

Line 72: Need better word than ‘proper’

**Reply:** The manuscript has been modified based on reviewer’s comments. We introduced how ruminants are physically distinguish from monogastrics (Lines 50-51 “Ruminants (cattle, sheep, goat) are physically distinguishable from monogastric animals due to the presence of forestomach (rumen, reticulum, omasum)”), and the importance of rumen development (Line 61-63 Neonatal ruminants (does not yet chew the cud; pre-ruminants) undergo physiological changes in the rumen before they can solely depend on fibre-rich diets (Heinrichs, 2005).”).

Line 77: pre-ruminant terminology doesn’t seem right.

**Reply:** Thanks for reviewer’s comment on the use of terminology. We have done a careful literature search to find the appropriate terminology for neonatal ruminants. Our search suggested that neonatal ruminants who do not engage in cud chewing is defined as pre-ruminants. We included the definition in the introduction (Line 62 “Neonatal ruminants (does not yet chew the cud; pre-ruminants) undergo physiological changes in the rumen before they can solely depend on fibre-rich diets”) to let the readers know why we used the term pre-ruminants in the present study. We also use this opportunity to provide clear information to a broader audience who are not familiar with ruminant biology.

Lines 84-85:  My main challenge with this paper is the lack of clarity over the techniques that were used n their totality.  The techniques defined in materials and methods do not match to the aims or abstract.

**Reply:** Following reviewer’s suggestion and the journal’s guideline, we have revised abstract section significantly. First, we shortened the abstract to meet the length limit (250 words), and also following reviewer’s suggestion we included brief statements of methods used to achieve the main results. We, however, included extra details at the end of the introduction on all methods and techniques. Please see Lines 80-86 “We employed next-generation sequencing of the rumen microbial metagenomes and rumen tissue transcriptomes (RNA-seq sequencing of host mRNAs and microRNAs) with an integrated bioinformatics approach to explore host-microbial interactions in the pre-ruminant rumen. Further, we evaluated the establishment and functionality of early rumen microbiota via quantification of active microbial densities (RNA-based) and VFAs (acetate, butyrate, propionate, branched-chain FAs) production.”

Materials and methods

This section needs some improvement. It seems you also completed metataxonomical analysis and qPCR but this isn’t mentioned in the abstracts or aims section of introduction.

**Reply:** The manuscript has been revised to clarify the materials and methods with details within text. Please see our detailed replies above under abstract and introduction.

We have carefully revised the methods to include details for all methods used. For example, we included how many animals were used for each analysis. Also, we changed titles to better represent the methods used and the objective of each analysis (Lines 480-481 *Profiling content-associated microbiome using whole genome based microbial shotgun metagenomics*; Line 536 *Profiling rumen transcriptome using RNA-seq*). Details on transcriptome data analysis (Lines 543-547, 560-570), integrated network analysis (Lines 584-598) were also added to the revised manuscript to increase clarity of methods used.

In general, the materials and methods section needs a diagram outlining the sampling and analysis conducted as it’s very confusing throughout the paper.

**Reply:** Thank you reviewer’s suggestions to improve the clarity of the manuscript. We have included a flow chart (Fig. 6) to present methods in details along with the revised text.

Transcriptome profiling. Suggest separate miRNA section from total transcriptome section so it makes understanding easier.

**Reply:** miRNAome expression data has been reported in Liang et al., 2016 PLoS ONE. We have added information on source of miRNA data (Lines 571-574 “MicroRNAome data of the same neonatal calves profiled using RNA-seq was obtained from our previously published work Liang et al., (2014). All expressed miRNAs (CPM > 1 in at least one sample) were used to further explore their regulatory mechanisms behind the host-microbial interactions in the developing rumen.”). Also, miRNA data was used in a network analysis similar to transcriptome (details can be found in Lines 584-598; Lines 608-611). In the revised version we included this new section (Lines 584-598) to present common methods used for both transcriptome and microRNAome.

Also, I’m confused, would the total metagenomic sequencing not have revealed sequences for non-bacterial microbes? Also for the tissue RNA sequences would this not have contained microbial metatarnscriptome sequences? Need to increase clarity running throughout paper.

**Reply:** Metagenomic sequences indeed contained host genes because there is trace of host DNA existing in the rumen contents due to the host cell death. In our analysis, host genomes were firstly removed before we perform functional and taxonomic assignment (Lines 497-500 “Artificial replicates, host (bovine) DNA and low-quality (Phred score < 25) sequences were removed from the raw data, and the remaining good-quality sequences were used to assign the taxonomy and functions.”). Following the taxonomic assignment, we only observed bacteria, fungi, and viruses in the rumen microbiome at birth (0-day). While, rumen contents of older calves (1 week, 3 week, 6 week) were colonized by archaea and protozoa as well from the first week of life other than bacteria, fungi, and viruses. This information is presented in results (Lines 96-98 “No archaea and protozoa were detected in the calf rumen at birth, while fungi and viruses together accounted for ~0.1% of total identified rumen microbiota (Supplementary Figure 1).” and Lines 116-118 “Rumen microbiome of pre-weaned calves (1-week, 3-week, 6-week) was colonized by bacteria, archaea, protozoa, fungi and viruses (Supplementary Figure 1), while bacteria remained predominant.”) with Supplementary Figure 1. We have also added a brief discussion on the establishment of archaea and protozoa to the revised manuscript (Lines 332-342).

We agree with reviewer that tissue RNA-sequencing can generate information for both host and microbes attached to the tissue. However, in this study, the host transcriptome data were generated based on enriched host mRNAs. During the library preparation, we enriched Eukaryotic mRNA with oligodT beads using TruSeq RNA Sample Preparation v2 kit. Further, only the transcripts mapped to bovine genome were considered as host genes and included to the analysis.  To increase clarity of the data presented, more details have been added to the methods section (Lines 539-540 “to enrich poly-A tailed host mRNA with oligodT beads”; Lines 543-544 “and only the reads mapped to bovine genome were used for further analysis.”). Methods to use host function enrichment were also added to the revised version in details (Lines 560-570).

Results

Lines 138-142: I could only see Phylum level data in the supplementary data?

**Reply:** Yes, we only reported phylum level data for tissue attached bacterial community. In the revised version, we decided to remove the results on epimural bacteria due to limited sample size and to make the manuscript more focused. Please see our above detailed replies to reviewer 1’s comments (under Line 242).

Line 170: metagenomic not metagenomics

**Reply:** The manuscript and the figures have carefully revised. See our detailed reply under abstract Line 22.

Line 180: This is Figure 1G not 1E, but there doesn’t seem to be a 1F? Need to check figure numbering. Also, there are no apparent microbes in the papillae images?

**Reply:** The manuscript and the figures have carefully revised. It seems we have mislabeled the figure panels. Figure legends and figure 1 panels have been revised to present and cite appropriate figures.

For the reviewer’s comments on “there are no apparent microbes in the papillae images”; the regular staining approach we used was not successful in visualizing tissue attached microbiota. To detect such population visually, SEM is required, and unfortunately, we did not collect samples for SEM at the time. However, we are in the process to work on tissue attached microbiota from other projects by collection of samples for SEM. Hopefully, such data can also be used to understand the role of epimural bacteria on rumen development (which we removed from the revised version due to the lack of data and absence of strong correlations following considering reviewer 1 comment but discussed in the discussion Lines 431-441).

Discussion

Line 323: what do you mean specifically when you talk about rumen transcriptome? Host? Microbial?

Don’t refer to figures/tables in discussion, discuss data in a more holistic manner.

**Reply:**  Here we referred to the studies that only focused on host. We have revised the manuscripts through to clearly indicate which host and microbial data respectively. In the revised version we stated “RNA-seq based profiling of host transcriptome has widely been studied in cattle to understand the changes occur in the rumen tissue with weaning, age, diet, and metabolic disorders at the molecular level of the system biology” (Lines 343-345).

Thanks for the suggestion to improve the discussion. In the revised version, we did our best to critically build the discussion in a holistic manner. We did re-think of a better way to present the summary of integrated omics approach and realized we can use a better summary figure (which Figure 5 cited in discussion and conclusion) to help readers to follow our discussion without going back to results. This figure was also used to introduce the phenomenon of bacteria-driven and ontogenic mechanisms (which is one of our main conclusions) of early rumen development while summarizing significant findings of the integrated omics approach. After considering reviewer’s comment we thought this modified Figure 5 will be useful to present the discussion with critical insights.

Conclusions

Remove Figure 5.

**Reply:**  Figure 5 was used to introduce the phenomenon of bacteria-driven and ontogenic mechanisms of early rumen development, which is one of our main conclusions. After considering reviewer’s comment we modified the Figure 5 to present the summary in a holistic manner and decided to keep it in the revised version. We hope the reviewer will understand our intention of using Figure 5.

**Second round of review**

**Reviewer 1**

*The authors have revised the manuscript according to my suggestions.*

**Reviewer 2**

*All replies significantly improve the manuscript and Figure 6 is very useful. I’m sure this paper will be well-cited.*
